# Pseudomonas aeruginosa Interstrain Dynamics and Selection of Hyperbiofilm Mutants during a Chronic Infection

**DOI:** 10.1128/mBio.01698-19

**Published:** 2019-08-13

**Authors:** Erin S. Gloag, Christopher W. Marshall, Daniel Snyder, Gina R. Lewin, Jacob S. Harris, Alfonso Santos-Lopez, Sarah B. Chaney, Marvin Whiteley, Vaughn S. Cooper, Daniel J. Wozniak

**Affiliations:** a Department of Microbial Infection and Immunity, The Ohio State University, Columbus, Ohio, USA; b Department of Microbiology and Molecular Genetics, University of Pittsburgh School of Medicine, Pittsburgh, Pennsylvania, USA; c Center for Evolutionary Biology and Medicine, University of Pittsburgh School of Medicine, Pittsburgh, Pennsylvania, USA; d School of Biological Sciences, Georgia Institute of Technology, Atlanta, Georgia, USA; e Emory-Children’s Cystic Fibrosis Center, Atlanta, Georgia, USA; f Department of Microbiology, The Ohio State University, Columbus, Ohio, USA; Mass General Hospital; Harvard Medical School; Yale University

**Keywords:** CRISPR-Cas, *Pseudomonas aeruginosa*, RSCV, Wsp, bacteriophages, biofilms, chronic infection, cyclic di-GMP, evolution, exopolysaccharide

## Abstract

Opportunistic pathogens establishing new infections experience strong selection to adapt, often favoring mutants that persist. Capturing this initial dynamic is critical for identifying the first adaptations that drive pathogenesis. Here we used a porcine full-thickness burn wound model of chronic infection to study the evolutionary dynamics of diverse Pseudomonas aeruginosa infections. Wounds were infected with a mixed community of six P. aeruginosa strains, including the model PA14 strain (PA14-1), and biopsies taken at 3, 14, and 28 days postinfection. Hyperbiofilm-forming rugose small-colony variants (RSCVs) were the earliest and predominant phenotypic variant. These variants were detected on day 3 and persisted, with the majority evolved from PA14-1. Whole-genome sequencing of PA14-1 RSCV isolates revealed driver mutations exclusively in the *wsp* pathway, conferring hyperbiofilm phenotypes. Several of the *wsp* mutant RSCVs also acquired CRISPR-Cas adaptive immunity to prophages isolated from the P. aeruginosa wound isolate (B23-2) that was also present in the inoculum. These observations emphasize the importance of interstrain dynamics and the role of lysogenic phages in the survival of an invading pathogen. Rather than being a side effect of chronicity, the rapid rise of RSCVs in wounds is evidence of positive selection on the Wsp chemosensory system to produce mutants with elevated biofilm formation capacity. We predict that RSCVs provide a level of phenotypic diversity to the infecting bacterial community and are common, early adaptations during infections. This would likely have significant consequences for clinical outcomes.

## Abstract (precis)

Bacteria adapt to infections by evolving variants that are more fit and persistent. These recalcitrant variants are typically observed in chronic infections. However, it is unclear when and why these variants evolve. To address these questions, we used a porcine chronic wound model to study the evolutionary dynamics of Pseudomonas aeruginosa in a mixed-strain infection. We isolated hyperbiofilm variants that persisted early in the infection. Interstrain interactions were also observed, where adapted variants acquired CRISPR-mediated immunity to phages. We show that when initiating infection, P. aeruginosa experiences strong positive selection for hyperbiofilm phenotypes produced by mutants of a single chemosensory system, the Wsp pathway. We predict that hyperbiofilm variants are early adaptations to infection and that interstrain interactions may influence bacterial burden and infection outcomes.

## INTRODUCTION

Chronic infections that persist despite extensive treatment are often attributed to biofilms, which are communities of adhered microorganisms encased in an extracellular polymeric substance (EPS) (1). Complicating chronic infections is the high likelihood that bacterial populations adaptively evolve, producing novel subpopulations with persistent phenotypes and increased fitness. When opportunistic pathogens leave an environmental reservoir to colonize a host, there are potentially many beneficial mutations available for selection in this environment. Defining this new fitness landscape and the evolutionary dynamics of pathogen adaptation to the host environment is critical to understanding infection pathology, as the emergence and selection of adapted variants in an infection are often associated with worsening clinical outcomes (2). Adapted variants are typically identified later in the infection, when eradication has become increasingly difficult. By monitoring the rise of adapted genotypes within pathogen populations, as the infection becomes established, we can understand the environment in which the pathogen adapts and predict the fitness constraints as a result of the adaptation. This may enable prediction of evolutionary pathways that could be targeted to improve treatment efficacy (3, 4).

Of interest to chronic infections are the emergence of rugose small-colony variants (RSCVs), which are associated with persistence (5). RSCVs are characterized by high fitness in biofilms, and they have been isolated from chronic infections (6, 7) and *in vitro*-grown biofilm experiments (8, 9). This suggests that there is strong selection for ecological diversification in both *in vivo*- and *in vitro*-grown biofilms. One of the most studied bacterial adaptive responses to chronic infection is that of Pseudomonas aeruginosa to the cystic fibrosis (CF) lung (10). CF patients exhibit airway abnormalities, where P. aeruginosa biofilms commonly colonize the mucus lining, and establish persistent pulmonary infections (11, 12). RSCVs are isolated from up to 50% of P. aeruginosa-positive CF sputum samples (6, 7). When isolated, RSCV frequencies range widely between patient samples, from 0.1 to 100% of the total population (7, 13). However, in comparison to the chronic pulmonary infections of CF patients, little is understood regarding P. aeruginosa adaptation in other common chronic infections, such as chronic wounds. Furthermore, understanding the selective forces that drive the emergence and frequency of RSCVs is important because of their association with hyperbiofilm-forming phenotypes (14, 15), increased tolerance to antimicrobials (13), and enhanced resistance to immunity (5).

The RSCV phenotype is commonly caused by mutations in pathways that lead to elevated cyclic diguanylate monophosphate (c-di-GMP) (2). c-di-GMP is a messenger molecule that signals the transition from planktonic to biofilm lifestyle in many bacteria (16). In P. aeruginosa, increased c-di-GMP, among many responses, leads to overproduction of exopolysaccharides, Psl and Pel, and matrix proteins (14, 17, 18). RSCVs with driver mutations in the Wsp (wrinkly spreader) pathway, originally identified in Pseudomonas fluorescens (19, 20), are commonly isolated from *in vitro*-grown biofilms, where approximately 70% of isolated P. aeruginosa RSCVs can be complemented by *wspF* in *trans* (17). Mutations in the Wsp pathway have been implicated in P. aeruginosa evolution in CF patients (21, 22). However, their importance in relation to other identified adaptive mutations and frequency in other chronic infections is unclear.

Despite our understanding of the divergent phenotypes of evolved variants, it is currently unclear which mutations and pathways experience selection in an infection, what new niches become occupied, and how these adaptations enable pathogen survival. It is often not feasible to monitor bacterial evolution from the onset of infection, as it is difficult to understand microbial dynamics in a clinical setting due to patient care, sampling difficulties, and cost. This has similarly proven challenging to monitor in a research setting, as there are few chronic infection models that mimic what is observed clinically. Furthermore, mutations also exist in the context of the larger microbial community. Chronic infection models typically address the adaptive traits of only a single founding clone, but susceptible individuals are continually exposed to different strains of opportunistic pathogens, particularly in the case of environmental organisms like P. aeruginosa. To address these challenges, we used a porcine full-thickness thermal injury wound model, which closely reflects human clinical chronic wounds (23–27), and is considered a general model for studying chronic infections established by bacterial biofilms (23, 25–27). Here, these wounds were inoculated with a coinfection of six P. aeruginosa strains, and we determine which strains persist, which produce adaptive colony variants, and identify the genetic and physiological pathways of adaptive evolution.

## RESULTS

### P. aeruginosa strains PA14-1 and PAO1-B11 were predominant in a mixed-strain chronic burn wound infection.

To determine the relative fitness of different P. aeruginosa strains and how the population evolves during chronic infection, we infected porcine full-thickness burn wounds with an inoculum consisting of approximately equal (but not identical) numbers of six different P. aeruginosa strains. Wounds were infected with two model strains (PA14-1 and PAO1-B11), three clinical isolates (B23-2, CF18-1, and S54485-1), and a water isolate (MSH10-2) (Table 1). Each strain had a unique nucleotide barcode introduced at the neutral Tn*7* site (Table 1). These strains share similar growth kinetics (see Fig. S1A and B in the supplemental material) and biofilm formation capacity (Fig. S1C). Wound biopsies were taken 3, 14, and 28 days postinfection (dpi) for bacterial quantification.

**TABLE 1:** Strains and plasmids used in this study

Strain or plasmid	Relevant genotype and/or characteristic(s)	Reference or source
P. aeruginosa strains used in the infection^*a*^		
PA14-1	Barcode CAAAAGGACA; Gent	
PAO1-B11	Barcode GTGTCGTGGG; Gent	
B23-2	Wound isolate; barcode GCCTATTGTG; Gent	Lubbock, TX
CF18-1	Nonmucoid CF isolate; barcode GTTACGTCAA; Gent	60
MSH10-2	Water isolate; barcode TATCAGATTT; Gent	60
S54485-1	UTI^*b*^ isolate; barcode TTAAACTAGG; Gent	60

P. aeruginosa strains		
PA14		
PA14Δ*wspF*	Clean *wspF* deletion	
PAO1		
PAO1Δ*wspF*	Clean *wspF* deletion (JJH356)	
PA14-1*attB*::*lacZ*	*lacZ* from miniCTX-*lacZ* introduced at the *attB* site	This study
RSCV-2 wt-*wspA*	Variant *wspA* replaced by the wild-type allele	This study
RSCV-1 wt-*wspA*	Variant *wspA* replaced by the wild-type allele	This study
RSCV-40 wt-*wspA*	Variant *wspA* replaced by the wild-type allele	This study

E. coli strains		
NEB5-α		NEB^*c*^
S17		NEB

Plasmids		
pEX18Ap	Gene deletion vector	47
pEX18Ap::*wt-wspA*	*wspA*-complementing construct	This study
pCdrA::*gfp*	CdrA promoter fused to *gfp;* Carb	31
pMH487	Empty vector for pCdrA::*gfp;* Carb	31
pJN2133	PA2133 cloned into pJN105; Gent	32
pHERD20T	Empty vector; Carb	56
pHERD2133	PA2133 from pJN2133 cloned into pHEDR20T; Carb	This study
miniCTX-*lacZ*	Tet	58

a All strains used in the infection are resistant to gentamicin. Barcodes are located at the Tn*7* site on the genome.

b UTI, urinary tract infection.

c NEB, New England Biolabs.

10.1128/mBio.01698-19.2 FIG S1 Phenotypic profile of P. aeruginosa strains used in the infection. (A) Metabolic activity of the six P. aeruginosa strains assayed for 16h at 37°C using a Biolog system. (B) Area under the curve (AUC) of the Biolog kinetic plot depicted in panel A. **, *P* value of <0.01. (C) Biofilms of the six P. aeruginosa strains were grown for 24 h at 37°C in a 96-well plate. Biomass levels were quantified by crystal violet staining. Data are depicted as mean ± SD (*n* = 3). (D) The frequency of each strain in the starting inoculum was determined by sequencing the strain-specific barcodes at the Tn*7* site. The proportion of each barcode was expressed as a percentage of the total sequence counts. Download FIG S1, PDF file, 0.1 MB. Copyright © 2019 Gloag et al. 2019 Gloag et al. This content is distributed under the terms of the Creative Commons Attribution 4.0 International license.

**FIG 1:**
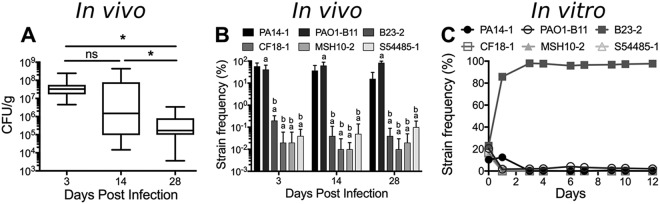
P. aeruginosa burden in a mixed-strain chronic burn wound infection. (A) Biopsies were taken from wounds at 3, 14, and 28 dpi. Biopsy specimens were homogenized and plated for CFU/gram, each plated in triplicate, with a minimum of four biopsy specimens taken from each wound. Significance was determined using a one-way ANOVA. ns, not significant; *, *P* value of <0.05. (B) Genomic DNA was isolated from homogenized tissue, and the strain-specific barcodes at the Tn*7* site were sequenced. A minimum of four biopsy specimens from each wound was sequenced. Significance was determined using a one-way ANOVA: a, *P* value of <0.05 compared to PA14-1; b, *P* value of <0.05 compared to PAO1-B11. (C) The six ancestor strains were competed in planktonic culture *in vitro* for 12 days. Three replicates were performed at each time point. For panels B and C, the proportion of strain barcodes were expressed as a percentage of the total sequence reads to determine the relative frequency of each strain.

Colony-forming unit (CFU) counts revealed that wounds remained colonized with approximately 10^5^ bacteria up to 28 dpi (Fig. 1A). To quantify the proportion of each strain over time, genomic DNA was isolated from biopsy tissue samples, and amplicons spanning the barcode were sequenced. As early as 3 dpi, PA14-1 and PAO1-B11 became the predominant strains in the infection, outcompeting the four other strains by orders of magnitude (Fig. 1B). To test whether PA14-1 and PAO1-B11 dominated in the infection due to host factors or due to interstrain competition, the six ancestor strains were competed together in planktonic culture *in vitro*, at similar starting frequencies used to inoculate the porcine wounds. Under these conditions, after 24 h, the wound isolate B23-2 outcompeted the other five strains (Fig. 1C). After 3 days, B23-2 remained the predominant strain, with PAO1-B11 the only other strain to be consistently detected, at approximately 2% of the population (Fig. 1C). This indicates that the predominance of PA14-1 and PAO1-B11 in the wounds was likely due to *in vivo*-specific factors (see Discussion).

### RSCVs were selected for during porcine chronic burn wound infections.

**FIG 2:**
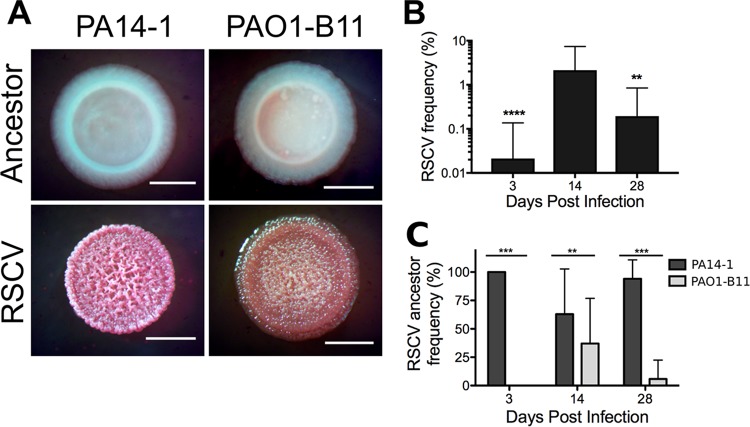
P. aeruginosa RSCVs isolated from porcine chronic burn wound infections. (A) Representative colony morphologies of RSCVs isolated from homogenized porcine burn wound tissue. RSCVs were plated on VBMM, and the colony morphology was compared to the ancestor strain (labeled). Bars, 2 mm. (B) Frequency of the total RSCV population isolated at each time point expressed as a percentage of the total P. aeruginosa population. Data are presented as mean ± standard deviation (SD). Significance was determined using a one-way ANOVA: **, *P* value of <0.01; ****, *P* value of <0.0001 compared to 14 dpi. (C) Frequency of the ancestor strain that the RSCVs evolved from, expressed as a percentage of the total RSCV subpopulation. Data presented as mean ± SD. Significance was determined using a Student’s *t* test: **, *P* value of <0.01; ***, *P* value of <0.001.

To identify functionally distinct mutants within the infections, we used colony morphology as an indicator (28, 29). Homogenized biopsy specimens were grown on Vogel-Bonner minimal medium supplemented with Congo red and brilliant blue dyes (VBMM). RSCVs were isolated from all three time points, with two subpopulations observed; one that had a pink, rugose phenotype and a second that had an orange, textured phenotype (Fig. 2A). The RSCV phenotype was stable across four passages on selective and nonselective growth media, consistent with the hypothesis that these variants were due to stable genetic mutations. In comparison, RSCVs were not isolated from the *in vitro* planktonic competition.

The total RSCV abundance in the wounds was quantified as a percentage of the total P. aeruginosa burden. RSCV frequency was low 3 dpi (0.02% ± 0.12%), peaked at 14 dpi, at approximately 2% (2.15% ± 5.25%), and declined at 28 dpi (0.19% ± 0.65%) (Fig. 2B). Despite their low prevalence, their rapid rise to detectable frequency allowed us to quantify the selective pressure acting on RSCVs in the wound, across a range of possible starting frequencies, according to equation 1. The selective coefficient (*s*) was >0.1, demonstrating that RSCVs were under strong positive selection (Table 2).

**TABLE 2:** Relative fitness, expressed as selection coefficient (*s*), of RSCVs from porcine burn wounds inferred for potential starting frequencies

Starting frequency (RSCV/ancestor)	*s* (mean ± SD) at the following days postinfection:		
	3	14	28
1:10^5^	0.585 ± 0.464	0.441 ± 0.126	0.18 ± 0.055
1:10^6^	0.618 ± 0.579	0.565 ± 0.177	0.229 ± 0.068
1:10^7^	0.651 ± 0.710	0.689 ± 0.238	0.278 ± 0.098
1:10^8^	0.684 ± 0.849	0.813 ± 0.304	0.327 ± 0.134

RSCVs were detected only from the model strains PA14-1 (the pink phenotype [Fig. 2A]) and PAO1-B11 (the orange phenotype [Fig. 2A]). RSCVs derived from PA14-1 were isolated across all time points, whereas PAO1-B11 RSCVs were isolated from 14 and 28 dpi. At the later two time points, PA14-1 RSCVs remained the predominant subpopulation (Fig. 2C).

### PA14-1 RSCVs contained driver mutations exclusively within the *wsp* pathway.

As PA14-1 RSCVs were the predominant evolved phenotype, we focused on this subpopulation for the remainder of this study. Whole-genome sequencing was performed on 27 randomly selected PA14-1 RSCVs to identify the mutation(s) accounting for the RSCV phenotype.

**FIG 3:**
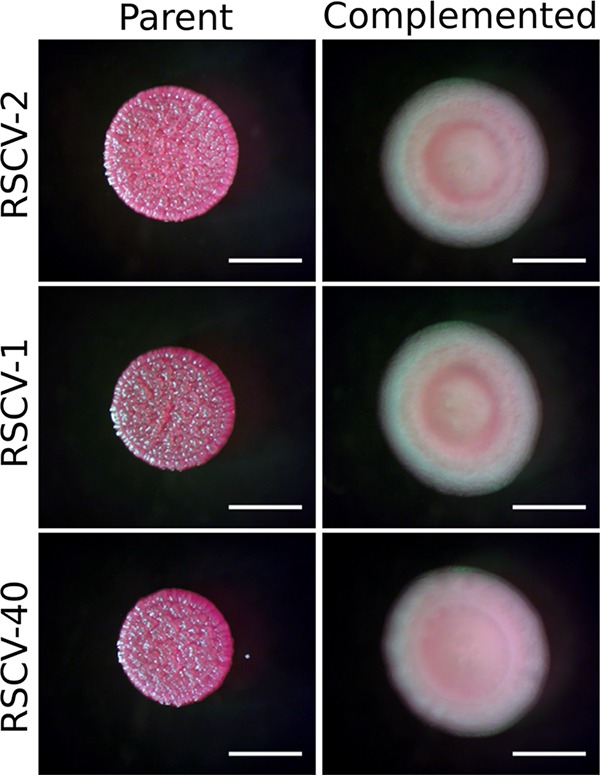
Complementation of the *wspA* Δ285-298 mutation. *wspA* was complemented in representative RSCVs by replacing *wspA* Δ285-298 with the wild-type allele on the genome. RSCV-2 was selected as a representative RSCV with the *wspA* driver mutation alone. RSCV-1 and RSCV-40 have the *wspA* driver mutation as well as Δ*pslE*-*pslO* and *wspD* secondary mutations, respectively. Parent and complemented RSCVs (labeled) were grown on VBMM, and colony morphologies were assessed. Bars, 2 mm.

We identified putative driver mutations exclusively in the *wsp* cluster, specifically, an in-frame, 42-bp deletion (Δ285−298 aa) in *wspA*, and a frameshift 5-bp deletion (V154fs) in *wspF* (Table 3). RSCVs with the *wspA* mutation were predominant across all time points, and *wspF* mutants were identified only at 14 dpi. Using RSCV-2 as a representative *wspA* mutant, we replaced the variant *wspA* with the wild-type allele using two-step allelic recombination to rescue the deletion. This resulted in the RSCV colony phenotype reverting to the wild-type phenotype (Fig. 3), demonstrating that the *wspA* Δ285-298 mutation was responsible for the RSCV phenotype.

**TABLE 3:** Mutations identified in PA14-1 RSCVs

Sample			Driver mutation			Secondary mutation	
Day	RSCV no.	Wound no.	Gene	Mutation^*a*^	Frequency (%)	Gene	Mutation
3	1	1	*wspA*	285-298del		*pslO-pslE*	Δ14,299 bp
	2	2	*wspA*	285-298del			
						*pslO-pslE*	Δ14,299 bp
	4	1	*wspA*	285-298del		PA14_13130/PA14_13140	TNN→TGC
						*fabI/ppiD*	TTC→TCC
							
Day 3 summary			*wspA*	285-298del	100		

14	3	4	*wspA*	285-298del			
	6	4	*wspA*	285-298del			
	7	4	*wspA*	285-298del			
	8	4	*wspA*	285-298del			
	9	4	*wspA*	285-298del			
	10	4	*wspA*	285-298del			
	12	4	*wspA*	285-298del		CRISPR–Cas1/hp^*b*^	+60bp
	13	1	*wspA*	285-298del		Glutamyl-tRNA reductase	L71L (CTG→TTG)
	14	4	*wspA*	285-298del		*fabI/ppiD*	TTC→TCC
	16	4	*wspA*	285-298del			
	17	4	*wspA*	285-298del			
	20	4	*wspF*	V154fs			
	24	1	*wspA*	285-298del			
	27	1	*wspA*	285-298del			
	28	2	*wspA*	285-298del			
	36	3	*wspF*	V154fs			
	37	3	*wspA*	285-298del			
	38	3	*wspF*	V154fs			
	86	3	*wspA*	285-298del		CRISPR–Cas1/hp	+60bp
							
Day 14 summary			*wspA*	285-298del	84.2		
			*wspF*	V154fs	15.8		

28							G→TT 585bp
						*wspD*	CT→AG 588-589bp
	40	1	*wspA*	285-298del			S197S (TCG→TCA)
						PA14_54090	A248V (GCG→GTG)
	42	3	*wspA*	285-298del			
	43	3	*wspA*	285-298del			
	45	3	*wspA*	285-298del			
	87	2	*wspA*	285-298del			
							
Day 28 summary			*wspA*	285-298del	100		

a Deleted amino acid residues are indicated. del, deletion; fs, frameshift.

b hp, hypothetical protein.

Some of the RSCVs also possessed secondary mutations (Table 3), possibly demonstrating further adaptation in the wound. Two *wspA* RSCVs from 3 dpi (Table 3; RSCV-1 and RSCV-4) underwent a 14,299-bp deletion that removed the remaining *psl* operon. PA14 naturally lacks Psl, since *pslA-pslD* are absent (30). In these two isolates, the remaining genes of the *psl* operon, *pslE-pslO* were deleted. In the RSCV-1 background, complementation of *wspA* reverted the RSCV colony phenotype to wild type (Fig. 3), indicating that deletion of the remaining *psl* operon did not influence the RSCV phenotype.

Evidence of additional adaptive mutations in the *wsp* cluster was detected on 28 dpi. RSCV-40, in addition to having the *wspA* Δ285-298 driver mutation, had three separate mutations in *wspD* which led to an early stop codon. However, as the mutations occurred at the *wspD* 3′ end, the WspD N terminus may still be expressed and functional (Table 3). In this isolate, complementation of *wspA* reverted the RSCV colony phenotype to wild type (Fig. 3), indicating that the *wspD* mutations did not influence the RSCV colony phenotype. However, this further points toward the strong selective pressure on the Wsp pathway in the chronic infection.

We were also interested in identifying how the PA14-1 non-RSCV population adapted to the infection and whether this population acquired mutations that did not result in divergent colony phenotypes. When we sequenced randomly selected PA14-1 non-RSCV isolates (23 total), relatively few had acquired chromosomal mutations (see Supplementary Results in Text S1 in the supplemental material; Table S1). These isolates had similar levels of biofilm formation and metabolic kinetics compared to the ancestor strain (see Supplementary Results in Text S1; Fig. S2), suggesting that the emergence of RSCVs was the main source of phenotypic diversification in the wounds.

10.1128/mBio.01698-19.1 TEXT S1 Supplemental Materials and Methods and Results. Download Text S1, PDF file, 0.2 MB. Copyright © 2019 Gloag et al. 2019 Gloag et al. This content is distributed under the terms of the Creative Commons Attribution 4.0 International license.

10.1128/mBio.01698-19.3 FIG S2 The PA14-1 non-RSCV population in the porcine wounds behave similarly to the ancestor strain. (A) Five non-RSCV colonies were randomly selected from each wound. The ancestor of each colony was identified and expressed as a percentage of the total number of colonies isolated from that time point. (B) Biofilms (24 h) were grown in 96-well plates, and biomass levels were quantified by crystal violet staining. Biomass levels are expressed as a percentage relative to PA14-1, which was set at 100%. ns indicates no significant difference. (C) AUC of Biolog kinetic curves. AUC are expressed as a percentage relative to PA14-1, which was set at 100%. Gray scale in panels B and C indicate different wounds. (D to F) Kinetic Biolog curves of representative non-RSCV PA14-1 isolates from each time point (labeled). Data are presented as mean ± SD (*n* = 3). *, *P* value of <0.05; **, *P* value of <0.01; ***, *P* value of <0.001. Download FIG S2, PDF file, 0.4 MB. Copyright © 2019 Gloag et al. 2019 Gloag et al. This content is distributed under the terms of the Creative Commons Attribution 4.0 International license.

10.1128/mBio.01698-19.8 TABLE S1 Mutations in non-RSCV PA14-1 population. Download Table S1, PDF file, 0.1 MB. Copyright © 2019 Gloag et al. 2019 Gloag et al. This content is distributed under the terms of the Creative Commons Attribution 4.0 International license.

### RSCVs isolated from wounds had elevated intracellular c-di-GMP levels.

**FIG 4:**
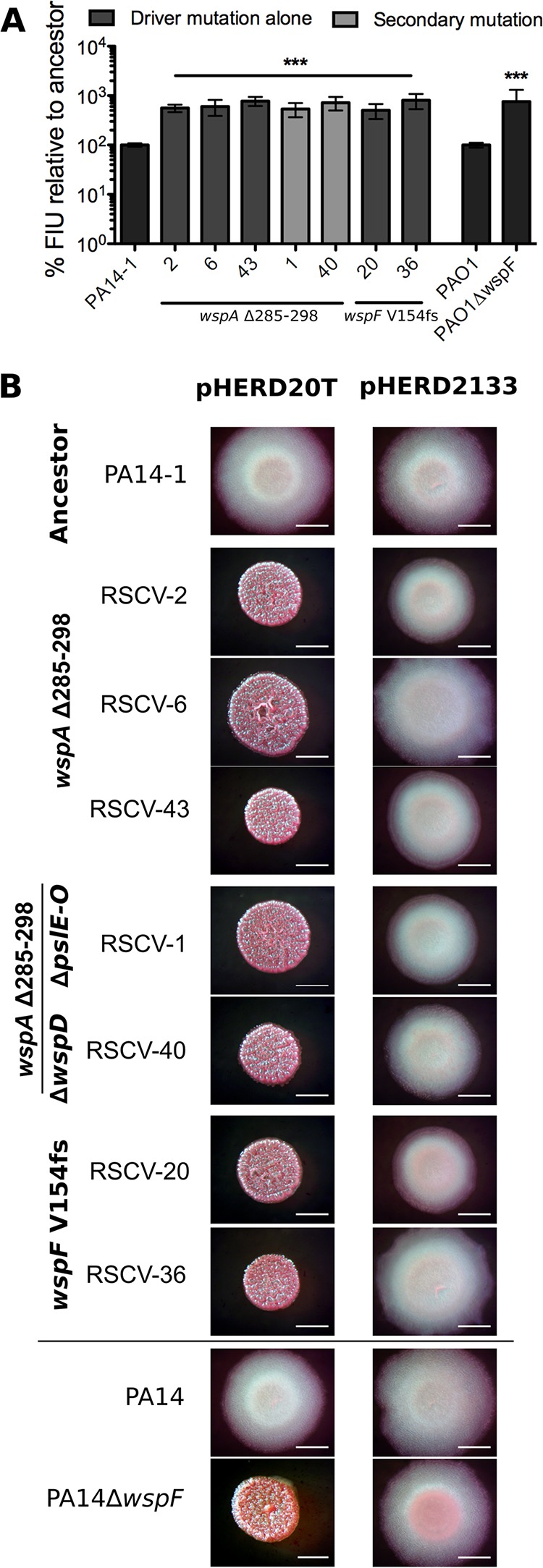
PA14-1 RSCVs have elevated intracellular levels of c-di-GMP which are responsible for the RSCV phenotype. (A) Green fluorescence was measured in representative RSCVs with the c-di-GMP reporter plasmid pCdrA::*gfp*. Increased GFP signal correlates to increased intracellular c-di-GMP levels. Fluorescence intensity units (FIU) of RSCVs were determined relative to the ancestor strain, which was set at 100%. PAO1Δ*wspF* and its isogenic parent PAO1 were used as controls. Data are presented as mean ± SD (*n* = 3). Significance was determined using a one-way ANOVA: ***, *P* value of <0.001 compared to the ancestor strain. (B) Colony morphology of representative RSCVs grown on VBMM plus 0.1% arabinose. pHERD20T is the empty vector. pHERD2133 has the PDE PA2133 cloned under an arabinose-inducible promoter. PA14Δ*wspF* and its isogenic parent, PA14, were used as controls. For both assays, representative RSCVs were selected. *wspA* mutants from each time point, RSCV-2, RSCV-6, and RSCV-43, were selected. Representative *wspA* mutants with secondary mutations were selected. RSCV-1 has the remaining *psl* operon deleted, and RSCV-40 has the additional *wspD* mutations. Representative *wspF* mutants, RSCV-20 and RSCV-36, were also selected. Bars, 2 mm.

As all PA14-1 RSCVs acquired driver mutations in the *wsp* cluster, we predicted that both the *wspA* and *wspF* mutations led to overproduction of c-di-GMP, resulting in the RSCV phenotype. To assess whether c-di-GMP levels were elevated, the pCdrA::*gfp* plasmid (31) was introduced into representative PA14-1 RSCVs. In this plasmid, the *cdrA* promoter, which is under c-di-GMP regulation, is fused to a promoterless *gfp* (31). As expected, the representative PA14-1 RSCVs showed increased levels of green fluorescence compared to the ancestor PA14-1, indicating that the RSCVs had elevated c-di-GMP levels (Fig. 4A). There were no differences in fluorescent signal between the RSCVs, regardless of the driver mutation or the presence of secondary mutations, indicating that the c-di-GMP levels were similar across the representative PA14-1 RSCVs (Fig. 4A). Introduction of a plasmid encoding the phosphodiesterase (PDE; enzyme that degrades c-di-GMP [16]) PA2133, under an arabinose-inducible promoter, into the representative PA14-1 RSCVs reverted the colony morphology to the ancestral type (Fig. 4B). The empty vector did not influence the RSCV colony morphology (Fig. 4B). Collectively, this indicates that elevated c-di-GMP levels were responsible for the RSCV colony phenotype, as has been observed in P. aeruginosa *wspF* mutants (32).

### Wsp-dependent RSCVs were more fit than the ancestor.

**FIG 5:**
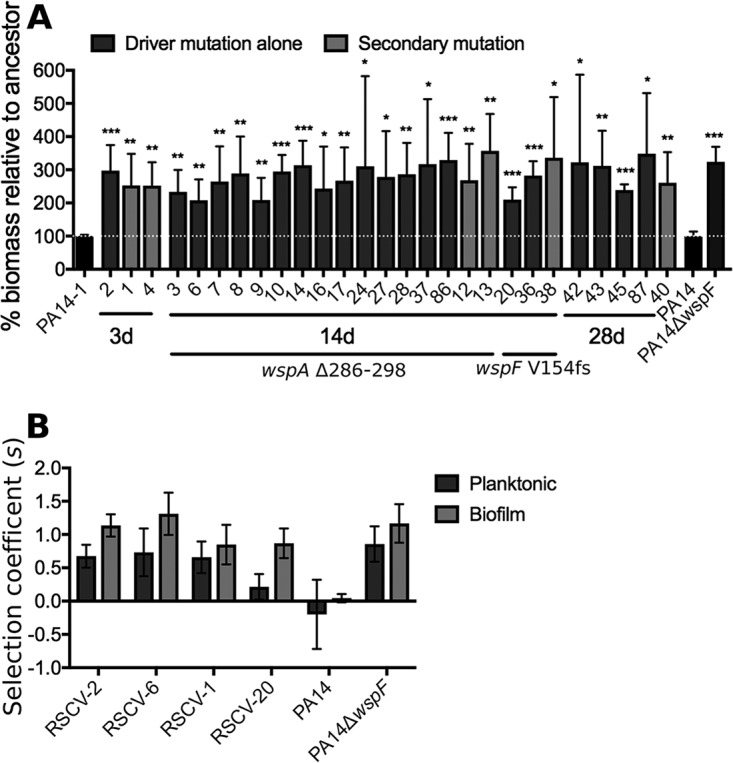
PA14-1 RSCVs have increased biofilm formation and fitness relative to the ancestor PA14-1. (A) PA14-1 RSCVs were grown in 96-well plates for 24 h. Biofilm biomass was stained and quantified by crystal violet. Biofilm biomass levels were expressed as a percentage relative to the ancestor strain, which was set at 100% for each replicate. Data are presented as mean plus SD (*n* = 3). Significance was determined using a one-way ANOVA: *, *P* value of <0.05; **, *P* value of <0.01; ***, *P* value of <0.001. (B) Fitness of representative PA14-1 RSCVs relative to the ancestor PA14-1. PA14Δ*wspF* and its isogenic parent PA14, compared to PA14-1, were used as controls. Strains were grown for 48 h as either a planktonic culture or biofilm, and selection of the RSCVs relative to PA14-1*attB*::*lacZ* was determined by calculating the selection rate according to equation 1. Data are presented as mean ± SD (*n* = 5).

RSCV colony morphology and elevated c-di-GMP are often associated with increased biofilm formation capacity. To investigate this possibility, biofilms were grown in microtiter plates for 24 h and stained with crystal violet. All 27 PA14-1 RSCVs displayed increased biofilm formation capacity compared to the ancestor PA14-1 (Fig. 5A). This increased biofilm formation was not due to elevated metabolic activity, as PA14-1 RSCVs had similar growth kinetics, except for an extended lag phase (Fig. S3).

10.1128/mBio.01698-19.4 FIG S3 PA14-1 RSCVs from the porcine wounds have an extended lag phase compared to the ancestor strain. (A) AUC of Biolog metabolic kinetic curves of all sequenced PA14-1 RSCVs, expressed as a percentage relative to PA14-1. PA14Δ*wspF* and its parent PA14 were used for comparison. *, *P* value of <0.01; #, *P* value of <0.001 compared to the ancestor strain. (B to E) Kinetic Biolog curves of representative PA14-1 RSCVs (labeled). Data are presented as mean ± SD (*n* = 3). Download FIG S3, PDF file, 0.3 MB. Copyright © 2019 Gloag et al. 2019 Gloag et al. This content is distributed under the terms of the Creative Commons Attribution 4.0 International license.

To determine the fitness of PA14-1 RSCVs relative to the ancestor strain *in vitro*, representative RSCVs were grown together with the ancestor PA14-1 tagged with *lacZ* at the neutral *attB* site (PA14-1*attB*::*lacZ*). Strains were grown either in planktonic culture or in a biofilm assay (33). The number of CFUs of each strain and the selection coefficient (*s*) were determined after 2 days as a measurement of fitness. All RSCVs tested were more fit than the ancestor under both modes of growth (Fig. 5B). Fitness in biofilm tended to be greater than in planktonic conditions, but these differences were not significant. The driver mutation did not appear to influence the selection rate, as RSCVs with either the *wspA* Δ285-298 or *wspF* V154fs had comparable fitness levels (Fig. 5B; RSCV-2 and RSCV-6 compared to RSCV-20). Furthermore, RSCV-1 (Δ*pslE*-*pslO*) had similar fitness levels compared to RSCV-2 and RSCV-6 (Fig. 5B). This suggests that loss of the remaining *psl* operon does not lead to an increased fitness advantage over the RSCV phenotype alone under these laboratory conditions.

### *In vivo* acquisition of immunity to viral infection through CRISPR expansion.

**FIG 6:**
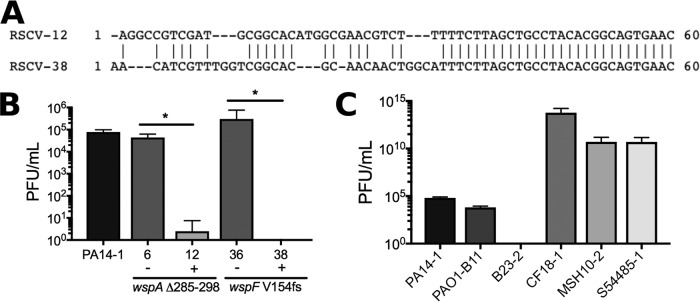
PA14-1 isolates RSCV-12 and RSCV-38 are resistant to infection by phage isolated from B23-2. (A) The 60-bp insertion sequence in the CRISPR array of RSCV-12 and RSCV-38. Prophages were isolated from B23-2, and plaque assays were performed to determine the level of phage infection for representative RSCV isolates (B) and the ancestor P. aeruginosa strains (C). RSCV-6 and RSCV-12 both have the same driver *wspA* mutation, while RSCV-36 and RSCV-38 have the same driver *wspF* mutation. The presence (+) or absence (−) of the CRISPR insertion is indicated. The driver mutation for each RSCV is labeled. Data are presented as mean plus SD (*n* = 4). Significance was determined using a Student’s *t* test: *, *P* value of <0.05.

Two of the PA14-1 RSCV isolates, RSCV-12 and RSCV-38, acquired a 60-bp insertion at the clustered regularly interspaced short palindromic repeat (CRISPR)–CRISPR-associated proteins (Cas) locus (Table 3). Both sequences inserted at the intergenic region (−1549/+271) between PA14_33350 (RS13600) and PA14_33370 (RS13605) at the genomic position 2,937,205. The last 28 bp of the inserted sequences were identical between the two isolates and aligned to the repetitive elements in the PA14 CRISPR array (CRISPR2 [34]) (Fig. 6A). However, the first 32 bp differed, indicative of CRISPR spacer sequences (Fig. 6A) that are specific to infective mobile genetic elements. A BLAST search of the inserted CRISPR spacer against the ancestor strains aligned to a prophage sequence in the B23-2 genome assembly, indicating potential phage infection across strains (see Supplementary Results in Text S1; Fig. S4).

10.1128/mBio.01698-19.5 FIG S4 Contig sequence from B23-2 with the protospacer sequence. There is currently no annotated sequence for B23. Therefore, the CRISPR spacer sequences were aligned against the contig sequences of the ancestor B23-2, which was sequenced along with the representative RSCVs. Protospacer for the CRISPR spacer in RSCV-12 (contig 107) (A) and RSCV-38 (contig 95) (B) were identified in two separate contigs (bold text). For the protospacer sequence in contig 95 (B), there was one base pair mismatch when comparing the CRISPR spacer to the protospacer (base pair that is not bold). After each protospacer, there is a conserved GG PAM motif (underlined) which is required for type 1-F CRISPR-Cas families which P. aeruginosa PA14 possesses. A BLAST search of the contig sequences identified CRISPR spacers in P. aeruginosa strains (Table S2) aligned to other protospacers in contig 107 (A), indicated by the red and blue text. Download FIG S4, PDF file, 1.6 MB. Copyright © 2019 Gloag et al. 2019 Gloag et al. This content is distributed under the terms of the Creative Commons Attribution 4.0 International license.

We therefore predicted that RSCV-12 and RSCV-38 would both be resistant to phages isolated from B23-2 due to CRISPR-Cas adaptive immunity. To test this, we grew B23-2 in mitomycin C and harvested the phage-enriched supernatant. P. aeruginosa strains were incubated with the phage lysate, and plaque assays were performed. RSCV-12 and RSCV-38 isolates were resistant to phage infection, with no plaques observed for RSCV-38, and only a single plaque in one replicate for RSCV-12 (Fig. 6B). Infection of RSCV-6 and RSCV-36 (CRISPR^−^), which have identical *wsp* driver mutations as RSCV-12 and RSCV-38 (CRISPR^+^), respectively, had similar levels of phage sensitivity compared to the ancestor PA14-1 (Fig. 6B). This indicates that the acquired CRISPR spacers in RSCV-12 and RSCV-38 produced immunity to phages isolated from B23-2. Each of the ancestor strains was assessed for phage susceptibility, and as expected, B23-2 was resistant to infection, whereas the remaining strains showed various levels of phage sensitivity (CF18-1 > MSH10-2, S54485-1 > PA14-1, PAO1-B11; Fig. 6C).

## DISCUSSION

Here we describe the rapid evolution of adaptive P. aeruginosa mutants with conspicuous colony phenotypes arising in a clinically relevant model of chronic infection. There is a consensus in the field that evolved variants arise in an infection as a consequence of adaptation over extended periods of time. However, we isolated hyperbiofilm-forming RSCVs from early stages of infection, which suggests that variants may evolve by positive selection more rapidly than originally appreciated. Therefore, contrary to current understanding, RSCVs may be a common, early adaptation during infections, and the selective pressures driving RSCV evolution may persist throughout these infections.

Interestingly, the competitive fitness of the six different P. aeruginosa strains differed *in vivo* and *in vitro*. In the wounds, both PA14-1 and PAO1-B11 outcompeted the remaining four strains, while in planktonic culture, B23-2 predominated (Fig. 1). This suggests that *in vivo* environmental factors may be responsible for driving the P. aeruginosa strain dynamics in the wound. We predict that *in vitro*, the prophage from B23-2 may be induced and responsible for driving strain competition and dynamics in planktonic culture. It would be interesting to explore this further and to determine, if so, why these dynamics might be dampened *in vivo*. This highlights the notion that fitness *in vitro* does not necessarily correlate to fitness *in vivo* and stresses the importance for using appropriate models when studying *in vivo* systems.

Because of their competitive superiority in the wound, RSCVs were recovered only from PA14-1 and PAO1-B11 (Fig. 2C). P. aeruginosa RSCVs were highly adaptive in the infection and in laboratory conditions (Table 2 and Fig. 5B). The estimated selection coefficients determined *in vivo* (*s *=* *0.180 to 0.813; Table 2) were up to five times greater than those identified in the Lenski long-term evolution lines (35), pointing toward the strong positive selection experienced by RSCVs in the wounds. In addition, similar to our results with PA14-1, Burkholderia cenocepacia variants containing *wsp* mutations isolated from an *in vitro* biofilm evolution assay had high selection coefficients (36). Together, these results suggest that *wsp* mutants experience significant positive selection both *in vivo* and *in vitro* and are broadly adaptive across different environments, where the biofilm lifestyle predominates.

Despite these strong selection coefficients, RSCVs remained at relatively low frequencies throughout the infection (Fig. 2B). This implies that RSCVs may be highly favorable when rare in the wounds, but less favorable when abundant, a process referred to as negative frequency-dependent selection. Negative frequency-dependent selection has been observed for evolved rugose variants of P. fluorescens (37–39). Niche competition (37, 38) and division of labor (39) with the ancestor strain drove the evolution of P. fluorescens rugose variants from static planktonic and colony growth, respectively. In both cases, diversification of the population was maintained by negative frequency-dependent selection (37–39). In the wound infections, the low-frequency RSCVs could facilitate the ancestor strain in colonizing and establishing biofilms in the wound by producing more EPS. This elevated EPS may enable persistence in an environment constantly exposed to stressors, including fluctuating antibiotics and immune defense (5). However, this may be a shortsighted evolutionary strategy for an opportunistic pathogen, like P. aeruginosa, with a significant environmental reservoir. The mutations in *wsp* are rarely reverted and constrain some of the phenotypic plasticity required for survival in fluctuating environments. Accordingly, we predict that in a more homogeneous fitness landscape, RSCVs at low frequencies could be enriched. As an example supporting this hypothesis, higher RSCV frequencies have been observed in CF patients under prolonged exposure to antimicrobials, particularly aerosolized antibiotics (6, 10).

Every sequenced PA14-1 RSCV acquired a driver mutation in the *wsp* cluster, demonstrating that in chronic wounds, the Wsp pathway specifically undergoes selection, and that *wsp* mutants may be more fit than other c-di-GMP-regulating pathways that confer the RSCV phenotype. The *wspA* Δ285-298 was the most common driver mutation and was isolated early in the infection (Table 3). There are two potential explanations for the rapid rise of this single *wspA* mutant in the infection. The first is that it may have been present in the initial inoculum at undetected levels. The second is that this region may be hypermutable owing to a direct repeat located at either side of the deletion (see Fig. S5A). We are currently unable to discern between these two scenarios; however, it is significant that the population rapidly diversifies in the wound due to strong positive selection of adaptive phenotypes provided by *wsp* mutations.

10.1128/mBio.01698-19.6 FIG S5 WspA deletions. (A) The 42-bp deletion in *wspA* is located between direct repeats. The gene sequence of PA14 *wspA* is shown. The 42-bp deletion (853-894bp) in the *wspA* mutants is underlined. Either side of this sequence is a direct repeat indicated in bold text. (B) Alignment of WspA deletions. Deletions in homologous regions of *wspA* have been observed in *in vitro*-evolved RSCVs; a 286-307aa deletion in P. aeruginosa PAO1 (MJK8), a 284-311aa deletion in *P. fluorescence* Pfl01 and a 307-313aa deletion in Burkholderia cenocepacia HI2424. The different domains of WspA were determined from Pfam analysis from the Pseudomonas Genome Database and the Burkholderia Genome Database. Domains are colored as follows: purple for the ligand binding domain or the four-helix bundle domain, green for the HAMP or linker domain, and blue for the MCP signaling domain. The region of the deletion in each protein is indicated in red. Download FIG S5, PDF file, 2.0 MB. Copyright © 2019 Gloag et al. 2019 Gloag et al. This content is distributed under the terms of the Creative Commons Attribution 4.0 International license.

Supporting the second theory is the observation that mutations in this *wspA* region also appear to be under selection to produce RSCVs in laboratory biofilms (Fig. S5B). The P. aeruginosa PAO1 RSCV isolate MJK8, which evolved during biofilm growth in a tube reactor (14), has an in-frame 66-bp deletion (Δ286–307aa) in the same region as the *wspA* Δ285-298 isolated here (17). P. fluorescens Pfl01, when grown as a colony biofilm, evolved RSCVs with driver mutations in *wspC*, *wspA*, and *wspE* (39). One of the *wspA* mutations was an in-frame 84-bp deletion (Δ284-311aa), again occurring in the homologous region (39). Finally, B. cenocepacia HI2424 RSCVs with *wspA* and *wspE* mutations were isolated from a biofilm bead evolution experiment (36). The majority of mutations identified were nonsynonymous single nucleotide polymorphisms (SNPs); however, one of the *wspA* mutations was an in-frame 21-bp deletion (Δ307-313aa), again in the homologous region (36) (Fig. S5B). Homology modeling of PA14 WspA suggests that the Δ285-298 occurs opposite the predicted methylation site (see Supplementary Results in Text S1; Fig. S6). We therefore predict that small deletions in the homologous region could alter how WspA is methylated/demethylated and ultimately lead to constitutive signaling and autoinduction of the Wsp pathway.

10.1128/mBio.01698-19.7 FIG S6 A 14-aa deletion in WspA is predicted to occur opposite the methylation site. (A) Schematic of WspA. The different domains of WspA were determined from the Pseudomonas Genome Database Pfam analysis. LBD, ligand binding domain or the four-helix bundle domain (3-182aa); HAMP, linker domain (213-261aa); SD, MCP signaling domain (348-505aa). The region of the 14aa deletion is indicated in red (285-298aa). (B) The WspA cytoplasmic domain amino acid sequence. The domains are indicated by the same colors as in panel A. The signaling domain contains two additional features, the kinase interacting subdomain, or “tip” domain in dark blue (382-420aa) and the predicted methylation site in navy blue (492-501aa). Both the heptad registers (reg) and the heptad number (h#) are labeled, with consecutive heptads indicated in alternating black and gray text. (C) Homology model of PA14 WspA modeled against the *T. maritime* MCP (PDB 3JA6) generated using SWISS-MODEL. Colors correspond to the domains indicated in panel B. The model spans 250-541aa of WspA. Download FIG S6, PDF file, 0.4 MB. Copyright © 2019 Gloag et al. 2019 Gloag et al. This content is distributed under the terms of the Creative Commons Attribution 4.0 International license.

In addition to driver mutations, some PA14-1 RSCVs also gained secondary mutations. Of particular interest was the Δ14,299 bp, which deleted the remaining *psl* operon (Table 3; RSCV-1 and RSCV-4). We predicted that this deletion might lead to increased fitness of the RSCVs, over the RSCV driver mutation alone. However, deletion of the remaining *psl* operon did not provide additional fitness benefits under the conditions tested. Therefore, the remaining *psl* operon (*pslE-pslO*) in PA14 may play a role outside Psl synthesis, which may have a fitness cost in the wound environment.

Additional secondary mutations of interest were the 60-bp insertions in the CRISPR-Cas array of RSCV-12 and RSCV-38 (Table 3 and Fig. 6A), which encoded resistance to phage(s) isolated from B23-2 (Fig. 6B). It has only recently been confirmed that the P. aeruginosa type I-F CRISPR-Cas system provides adaptive immunity to phages with a target protospacer (40). This is dependent on the presence of the correct protospacer adjacent motif (PAM) in the mobile genetic element (40). In support of this, both protospacers contain the type I-F CRISPR-Cas-specific GG PAM (Fig. S4). Furthermore, B23-2 contig 107 contained two additional protospacers to which CRISPR spacers in P. aeruginosa have been reported (Table S2 and Fig. S4A). Of interest was the observation that PA14 already contains a CRISPR spacer identical to a protospacer in contig 107 (Table S2 and Fig. S4A), possibly indicating that PA14 had already been exposed to the prophage in B23-2. However, the ancestral PA14-1 was still sensitive to infection (Fig. 6B and C), presumably due to the incorrect PAM (Fig. S4A). This highlights the importance of insertion of the correct CRISPR spacer in mediating phage immunity. This is only the second report of CRISPR-Cas acquired immunity in P. aeruginosa strains (40), and to our knowledge, this is the first report of CRISPR-Cas adaptive immunity acquired in an infection.

10.1128/mBio.01698-19.9 TABLE S2 CRISPR spacers in P. aeruginosa strains with a protospacer aligning to contig 107 in B23-2. Download Table S2, PDF file, 0.01 MB. Copyright © 2019 Gloag et al. 2019 Gloag et al. This content is distributed under the terms of the Creative Commons Attribution 4.0 International license.

Our data indicate that P. aeruginosa experiences strong selective forces in chronic infections, from interstrain phage predation to increased biofilm or aggregate production, and in response rapidly evolve during the initial stages of infection. Parallel evolution of the *wsp* pathway was observed across all time points, suggesting that *wsp* mutants are early, highly beneficial adaptations to infection. This also indicates that the Wsp system is a potent pathway under selection in chronic infections to produce hyperbiofilm variants. This is despite the availability of other c-di-GMP-regulating pathways to produce RSCVs, both *in vivo* and *in vitro* (18, 41–45). We predict that RSCVs produced through the *wsp* pathway may be an adaptation common to chronic infections, and developing therapies that target the RSCV subpopulation or prevent their emergence could be transferrable across these infections.

## MATERIALS AND METHODS

### Bacterial strains and plasmids.

Bacterial strains and plasmids used in this study are detailed in Table 1. Complementation constructs were made using Gibson Assembly (New England Biolabs [NEB]) (46). Primers used to create the constructs are detailed in Table S3 in the supplemental material. Constructs were incorporated into the P. aeruginosa genome using two-step allelic recombination as previously described (47).

10.1128/mBio.01698-19.10 TABLE S3 Primers used in this study. Download Table S3, PDF file, 0.02 MB. Copyright © 2019 Gloag et al. 2019 Gloag et al. This content is distributed under the terms of the Creative Commons Attribution 4.0 International license.

### Porcine full-thickness chronic burn wound model.

Swine were housed and studied according to the Ohio State University IACUC-approved protocols. Porcine full-thickness chronic burn wounds were established as previously described (23). Briefly, two pigs were subjected to thermal injury, giving six full-thickness burns per pig and covered with impermeable wound dressings. Wounds were infected 3 days after injury with equal amounts of six different P. aeruginosa strains to achieve a final 250-μl inoculum at 10^8^ bacteria (1.6 × 10^7^ each for a total of 1 × 10^8^), which was spread over the wounds and allowed to air dry before the wound dressing was reapplied. Wounds were infected with P. aeruginosa strains PA14-1, PAO1-B11, B23-2, CF18-1 (GenBank identifier [ID] NZ_KI519281), MSH10-2 (GenBank ID NZ_KE138672), and S54485-1 (GenBank ID NZ_KI519256). Prior to infection, each strain had been tagged with a unique barcode at the Tn*7* site on the genome (see Supplementary Methods in Text S1 in the supplemental material; Table 1).

Wound healing was monitored 3, 14, and 28 days postinfection. At each time point, four to eight 8-mm punch biopsies were taken from two wounds on each pig (four wounds for each time point). Biopsy specimens were homogenized in 1 ml phosphate-buffered saline (PBS) and plated on *Pseudomonas* isolation agar (PIA) supplemented with 100 μg/ml gentamicin for CFU/gram tissue. To screen for the emergence of adapted P. aeruginosa variants, homogenized tissue was also plated onto adjusted Vogel-Bonner minimal medium (VBMM) (see Supplementary Methods in Text S1) supplemented with 100 μg/ml gentamicin. Colony morphology variants were passaged onto PIA followed by two rounds on Luria agar (LA), before being plated back onto VBMM (without antibiotics) to confirm that the variant phenotype was a result of a stable mutation. Confirmed colony variants were stored at −80°C.

The selection of RSCVs in the wound was determined by calculating the selection coefficient (*s*) according to equation (1) (48)

(1) $$s=\frac{\text{ln}\left(\frac{M_{x}}{M_{0}}\right)-\ln\left(\frac{W_{x}}{W_{0}}\right)}{T_{x}}$$

where *T_x_* is day *x* and *M* and *W* are the number of mutant and wild-type cells, respectively, at day *x* and 0.

### Sequencing and analysis.

To determine the frequency of each strain across the infection, strain-specific barcodes were amplified and given Illumina sequencing adapters as detailed in Supplementary Methods in Text S1. Library sequencing pools were sequenced on NextSeq and MiniSeq High Output SE75 runs at the Petit Institute Molecular Evolution Core Facility at Georgia Institute of Technology. Analysis script is available on GitHub (https://github.com/glew8/Barcode_Sequencing). Briefly, FastQC 0.11.7 and MultiQC v1.5 were used to confirm sufficient sequencing quality (49, 50). Cutadapt 1.13 was used to select sequences with the insert sequence and parse reads containing each barcode, which were counted using egrep (51).

To identify the ancestor strain that the isolated RSCVs evolved from, colony PCRs were performed using ancestor strain-specific primers. Forward primers contained the unique barcode used to tag each ancestor strain at the Tn*7* site. Primers are indicated in Table S3.

To identify mutations, genomic DNA was isolated from colony variants using the DNeasy Blood and Tissue kit (Qiagen) according to the manufacturer’s protocol. Clonal DNA was sequenced on the Illumina NextSeq 500 at the University of Pittsburgh Microbial Genomics Sequencing center using a modified protocol for library prep using the Illumina Nextera kit (52). 2x151bp sequencing reads for selected isolates were trimmed and quality filtered using Trimmomatic v0.36 (settings: LEADING:20 TRAILING:20 SLIDINGWINDOW:4:20 MINLEN:70) (53). The reads passing quality filtering were then used for variant calling with the open-source program *breseq* v0.30.0 using default settings (54).

### Planktonic competition.

To compete the six ancestor strains *in vitro*, strains were grown independently overnight in supplemented M9 medium (42.2 mM Na_2_HPO_4_, 22 mM KH_2_PO_4_, 21.7 mM NaCl, 18.7 mM NH_4_Cl, 0.1 mM CaCl_2_, 1 mM MgSO_4_, and 11.1 mM glucose, supplemented with 20 ml/liter minimum essential medium [MEM] essential amino acids, 10 ml/liter MEM nonessential amino acids, and 1 ml each of trace mineral solutions A, B, and C [Corning catalog no. 25021-3Cl] [55]). Replicate tubes containing 5 ml supplemented M9 medium were inoculated with 50 μl of each overnight culture. Each day, for 12 days (∼80 generations total), 50 μl of culture was transferred to a new tube filled with 5 ml supplemented M9 medium. Populations were frozen on days 1, 3, 4, 6, 7, 9, 10, and 12. At each time point, genomic DNA was isolated and sequenced as described above for mutation calling. Three replicates were sequenced at each time point. All sequencing reads from isolates are deposited in NCBI SRA under Biosample accession numbers SAMN11956547 to SAMN11956595.

### c-di-GMP assays.

c-di-GMP reporter plasmid pCdrA::*gfp* and empty vector pMH487 (31) were electroporated into selected isolates. Overnight cultures were diluted to an optical density at 600 nm (OD_600_) of 0.1, and the cell pellets were resuspended in PBS. One hundred microliters were transferred to a polystyrene black 96-well plate (Corning). Cells were measured on a SpectraMax i3 plate reader (Molecular Devices) for OD_600_ and GFP fluorescence. Fluorescence was measured as fluorescence intensity units (FIU) using an excitation of 485 nm and emission of 535 nm. Four biological replicates, each with three technical replicates, were performed. The FIU values were normalized to OD, and the average autofluorescence from the empty vector control was subtracted for each strain. The FIU of RSCVs was determined relative to the ancestor strain, which was set at 100%.

The P. aeruginosa PDE PA2133 was shuttled from pJN2133 (32) as an EcoRI-XbaI fragment into pHERD20T (56), forming the new plasmid pHERD2133. This was electroporated into selected isolates. Colony morphology was assayed on VBMM with 0.1% arabinose.

### Biofilm assays.

Microtiter crystal violet assays were performed as previously described (57). Briefly, overnight cultures were diluted 1:10 into fresh media, and 100 μl was transferred into a 96-well round-bottom polyvinyl chloride (PVC) plate (Corning) and incubated for 24 h at 37°C in a humidified chamber. Biofilms were stained with 120 μl of 0.1% crystal violet for 30 min at room temperature. Crystal violet was extracted with 150 μl of 100% ethanol for 30 min at room temperature. One hundred microliters was transferred to a new plate, and OD_590_ was measured on a SpectraMax i3 plate reader (Molecular Devices). Three biological replicates, each with two technical replicates, were performed.

### Fitness assays.

The ancestor strain PA14-1 was tagged with *lacZ* at the *attB* site to generate the strain PA14-1*attB*::*lacZ*. Briefly, miniCTX-*lacZ* (58) was conjugated into PA14-1. The vector backbone was removed by electroporating pFLP2 (59), which was subsequently cured by growth on LA supplemented with 10% sucrose. Colonies were screened for tetracycline and carbenicillin sensitivity and blue selection on 5-bromo-4-chloro-3-indolyl-β-d-galactopyranoside (X-Gal).

Overnight cultures were grown in 5 ml tryptic soy broth (TSB) (30 g/liter TSB). For each competition, five replicate tubes were inoculated with 25 μl of overnight culture of both the RSCV and the marked ancestor (PA14-1*attB*::*lacZ*). Cultures were inoculated into tubes containing 5 ml of TSB either with a 7-mm polystyrene bead (biofilm) or without the bead (planktonic). After 24 h, 50 μl of culture was transferred to a second tube containing fresh TSB for the planktonic cultures, and for the biofilm cultures, the biofilm-coated polystyrene bead was transferred to a second tube containing an uninoculated bead. After a second round of 24-h growth, all competitions were diluted and plated for CFU counts onto tryptic soy agar plates solidified with 1.5% agar and supplemented with 80 mg/ml X-Gal. In order to assess biofilm CFU, the beads were sonicated in PBS with a probe sonicator for 10 s at 30% power, and the resulting supernatant was used for plating. The selection coefficient (*s*) was calculated according to equation 1.

### Prophage isolation and plaque assay.

To isolate prophages from B23-2, an overnight culture of B23-2 was diluted 1:100 and incubated for 30 min at 37°C with shaking at 200 rpm. Mitomycin C (0.5 μg/ml) was added to the culture, and the OD_600_ was measured. The culture was reincubated, and the OD_600_ was measured every hour. When the OD_600_ began to decrease, the cells were pelleted by centrifugation, and the supernatant was filter sterilized and stored at 4°C.

To determine the level of susceptibility of P. aeruginosa strains to the bacteriophage(s) isolated from B23-2, 100 to 200 μl of mid-log P. aeruginosa culture was incubated with 100-μl serial dilutions of bacteriophage lysate for 15 min at 37°C. The infection was added to 5 ml molten soft agar (LB solidified with 0.7% agar) supplemented with 10 mM CaCl_2_ and MgSO_4_. This was then poured over solidified hard agar (LB solidified with 1.5% agar), allowed to solidify, and incubated overnight. The number of resulting plaques was then counted, and the number of PFU/ml was determined.

### Statistical analysis.

Data are presented as mean ± standard deviation (SD). To determine whether data conformed to a normal distribution, a Shapiro-Wilk test was performed. All of the data sets were normally distributed except for the PFU/ml data (Fig. 6). For these data sets, means were compared using the nonparametric *t* test. All other comparisons were made using a one-way analysis of variance (ANOVA) with a Tukey’s post-hoc test and Student’s *t* test. Analyses were performed using GraphPad Prism v.5 (GraphPad Software). Statistical significance was determined using a *P* value of <0.05.

### Data availability.

The reference sequences for variant calling were acquired from NCBI’s RefSeq database (NC_002516.2 for PAO1, NC_008463.1 for PA14). All sequencing reads from isolates are deposited in NCBI SRA under Bioproject number PRJNA491911 and Biosample accession numbers SAMN10101410 to SAMN10101459.
