# Supplementary figures and images for: Pseudomonas aeruginosa Interstrain Dynamics and Selection of Hyperbiofilm Mutants during a Chronic Infection
Source: mBio. 2019 Aug 13;10(4):e01698-19. doi: 10.1128/mBio.01698-19 (PMC6692513; doi:10.1128/mBio.01698-19)

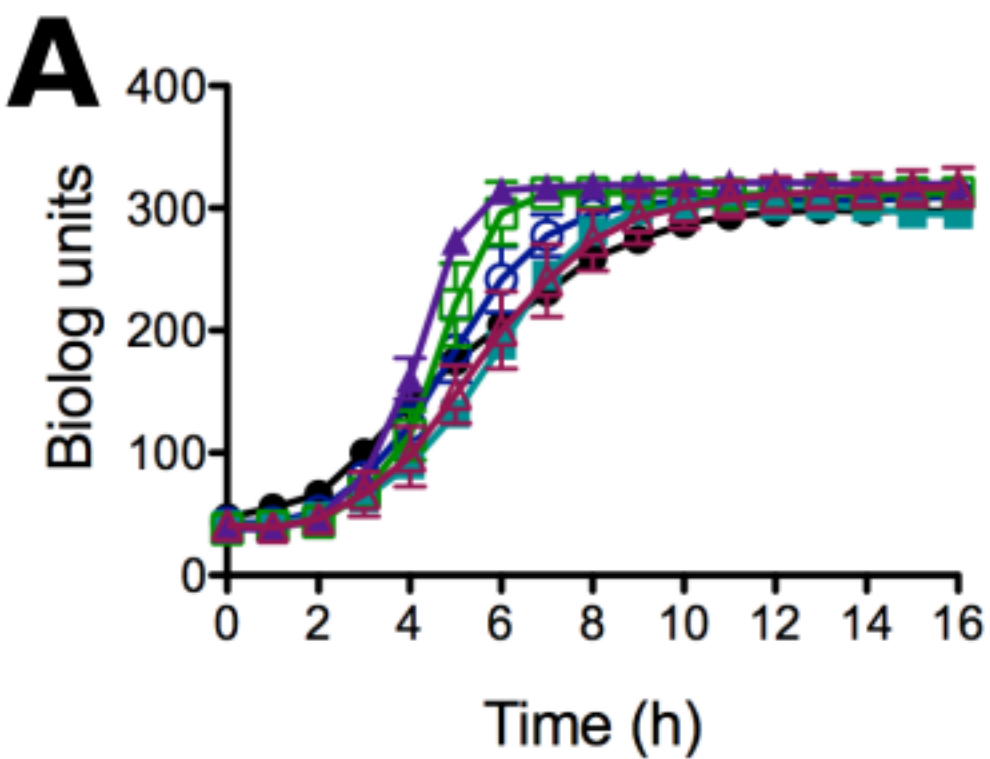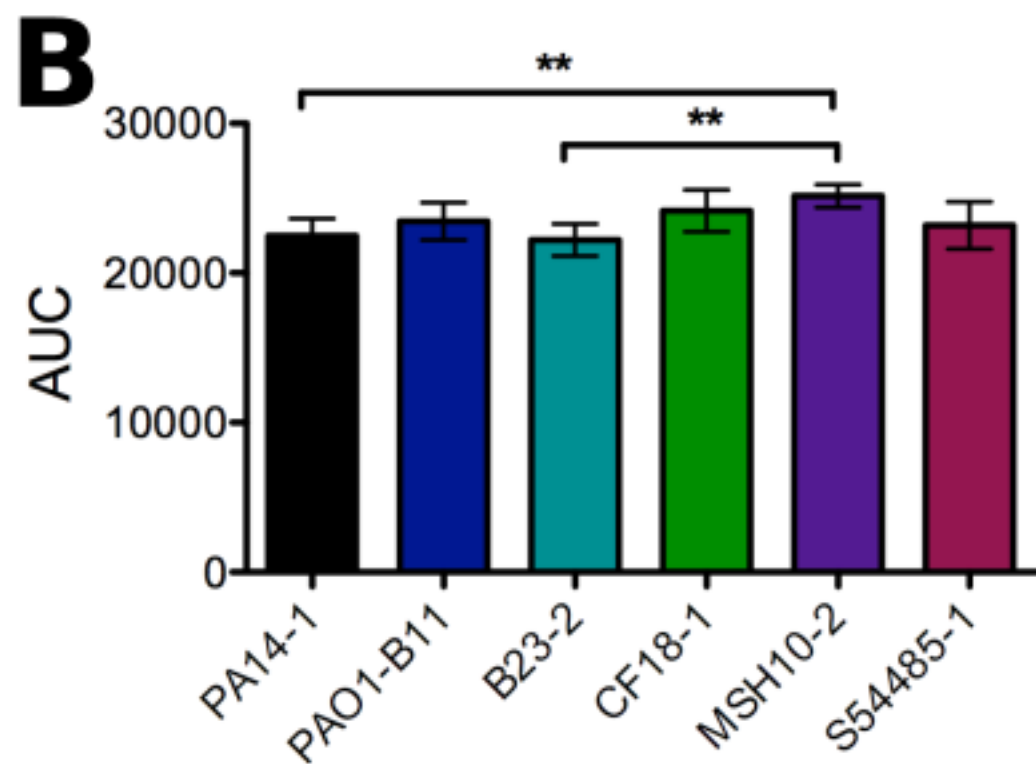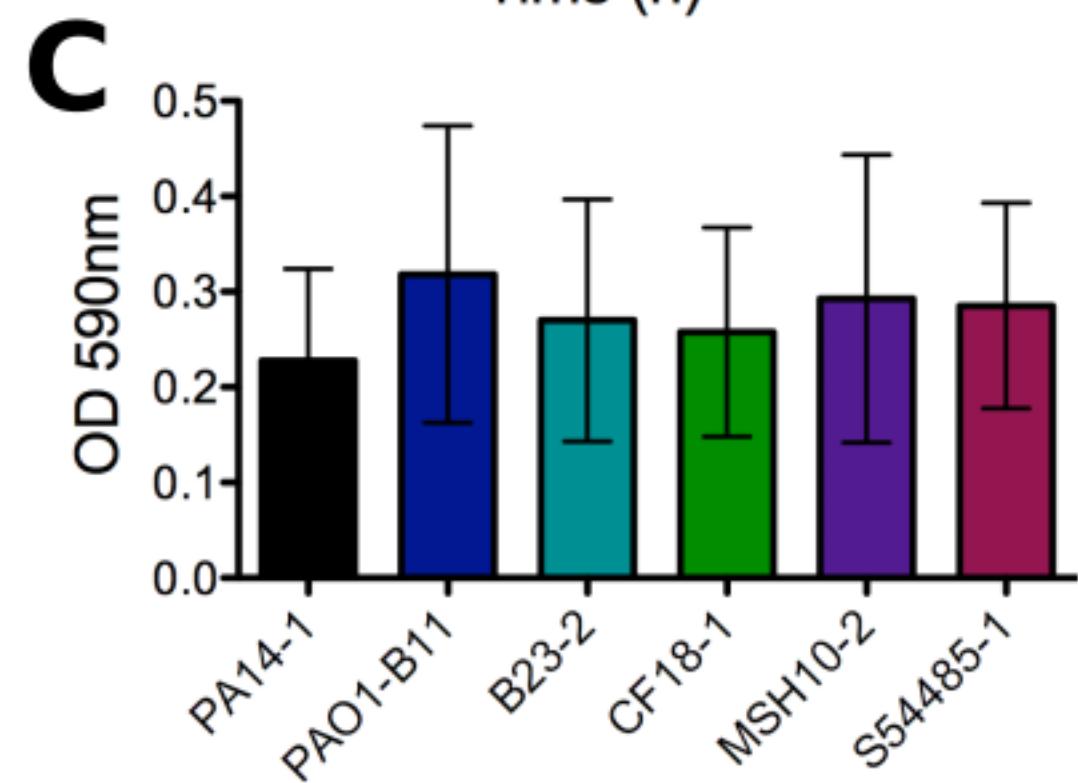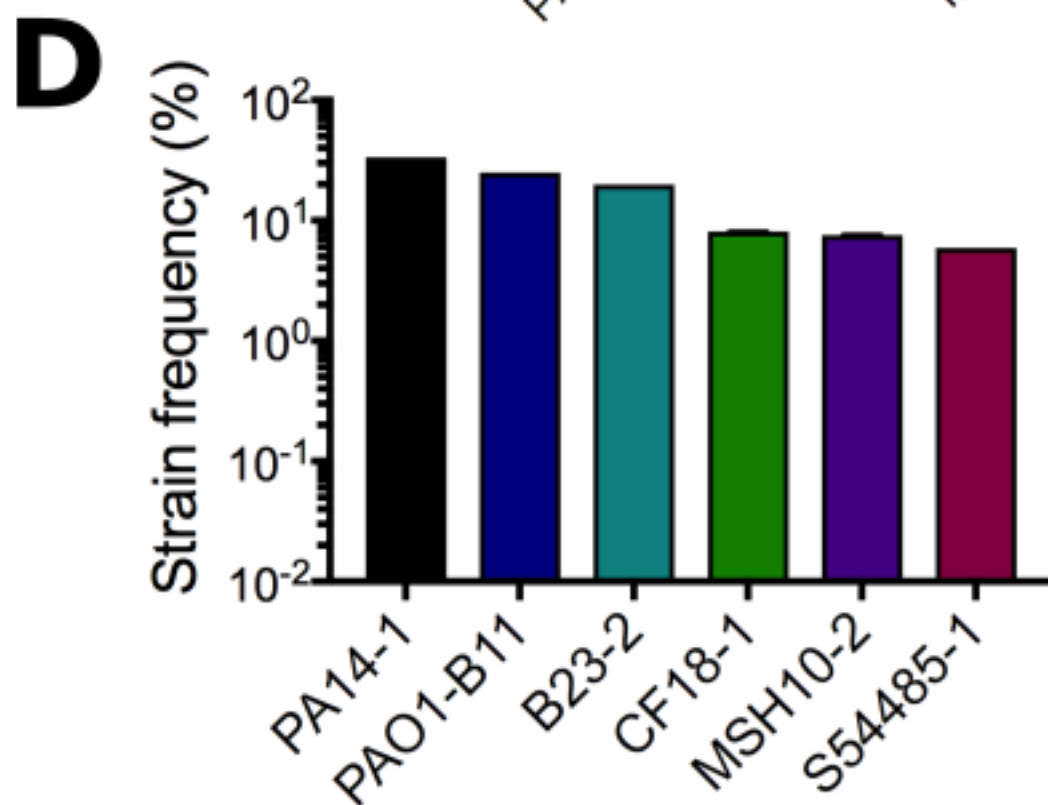

Supplement: FIG S1 [file mBio.01698-19-sf001.pdf]

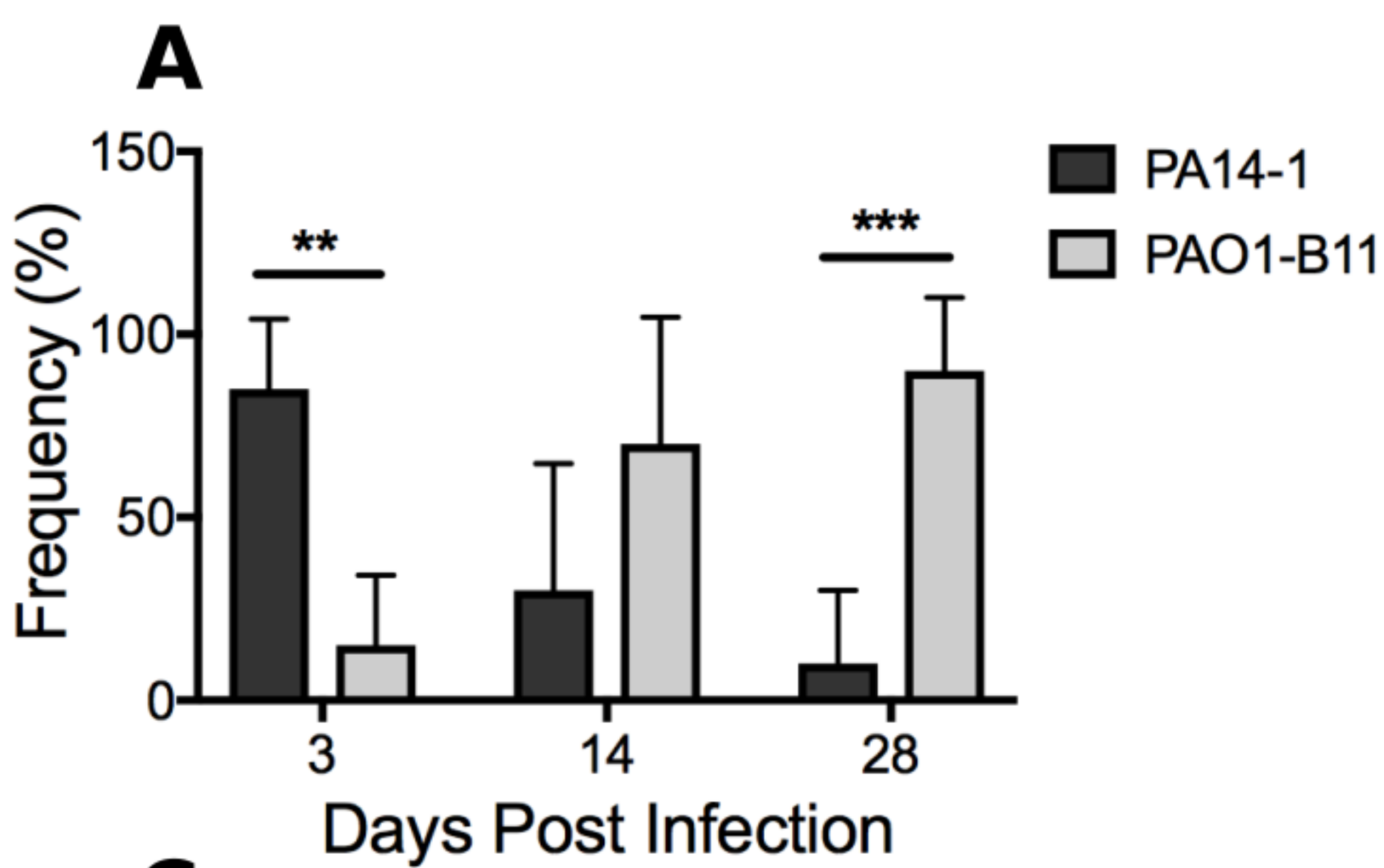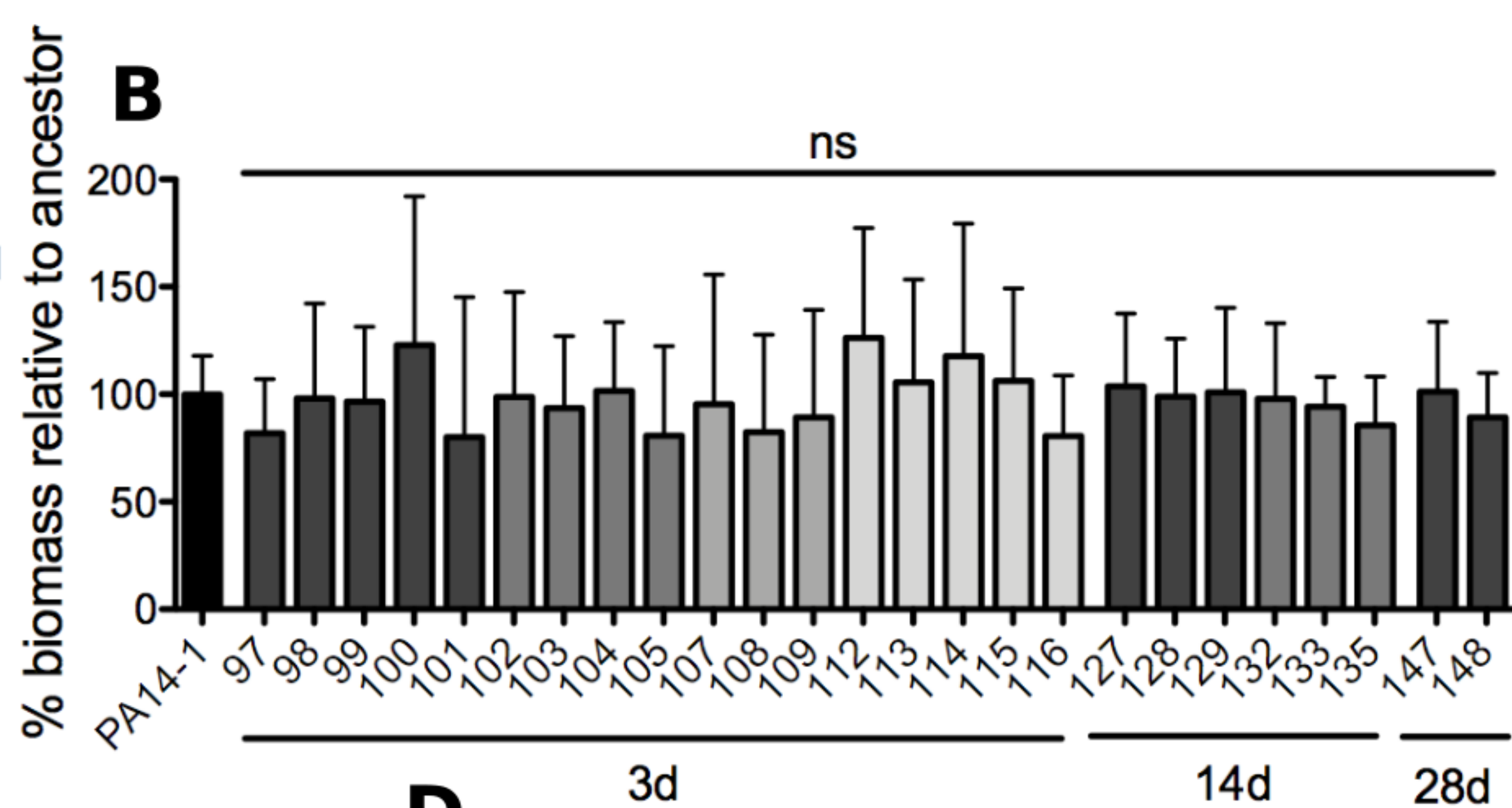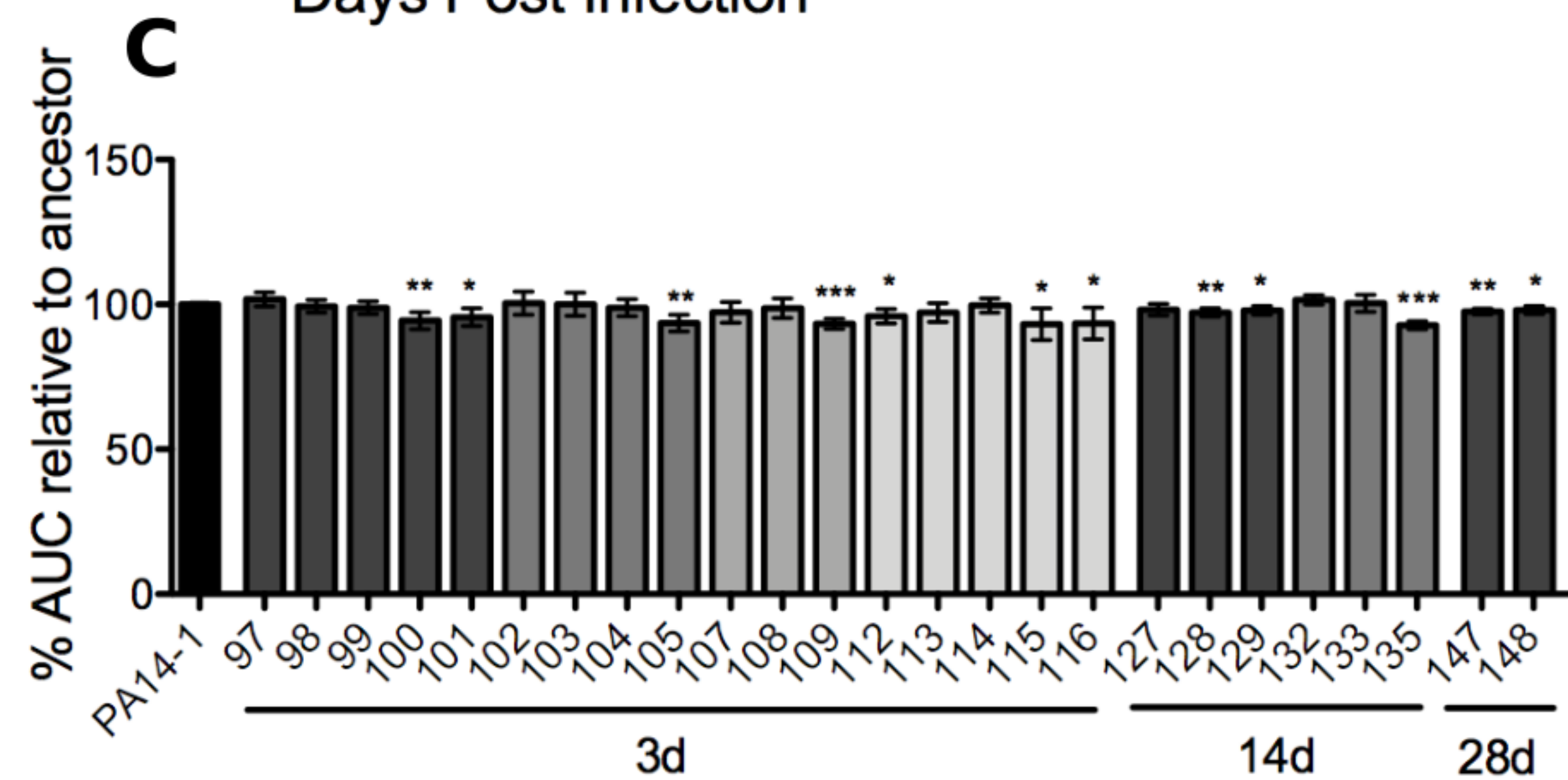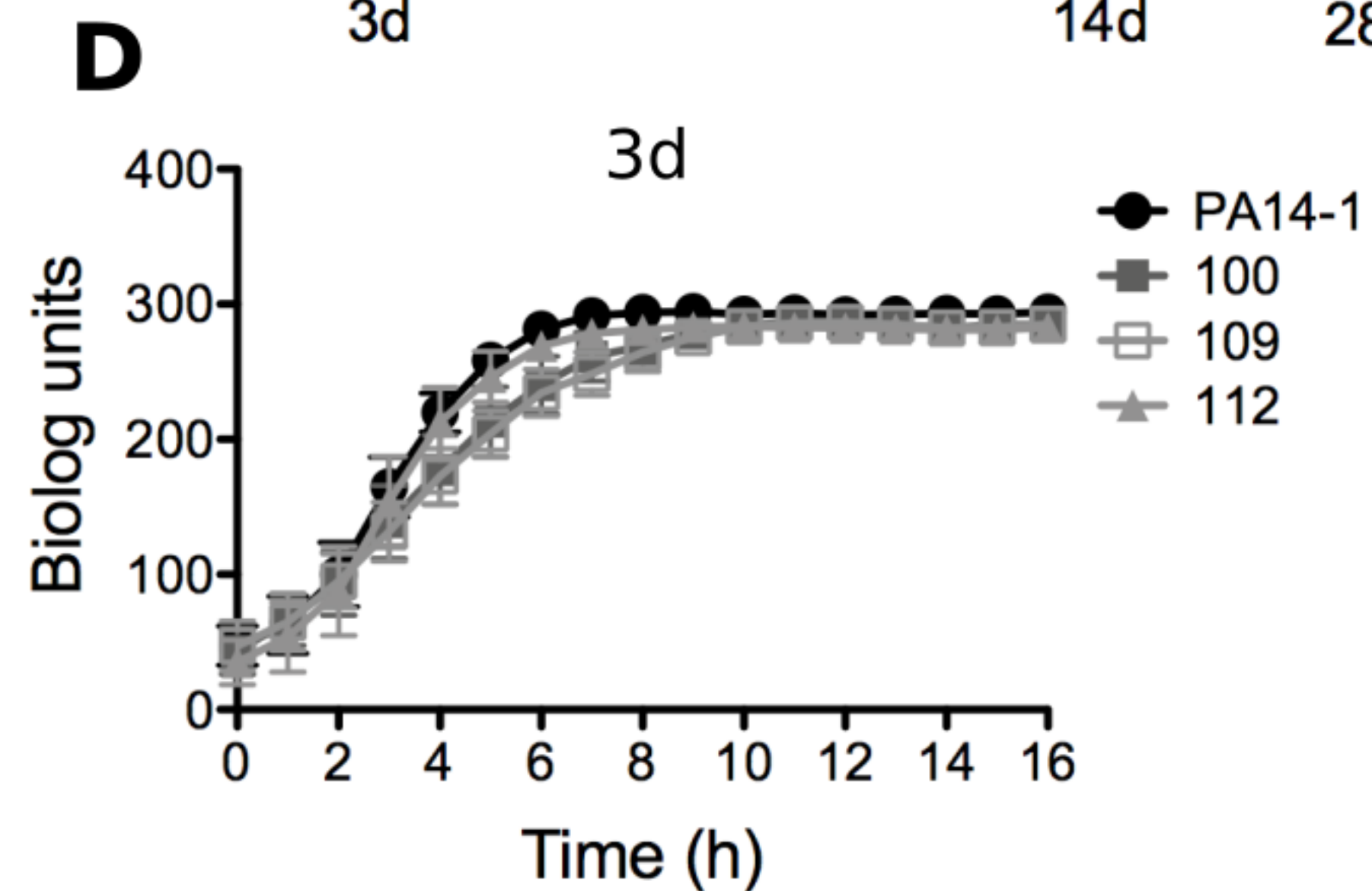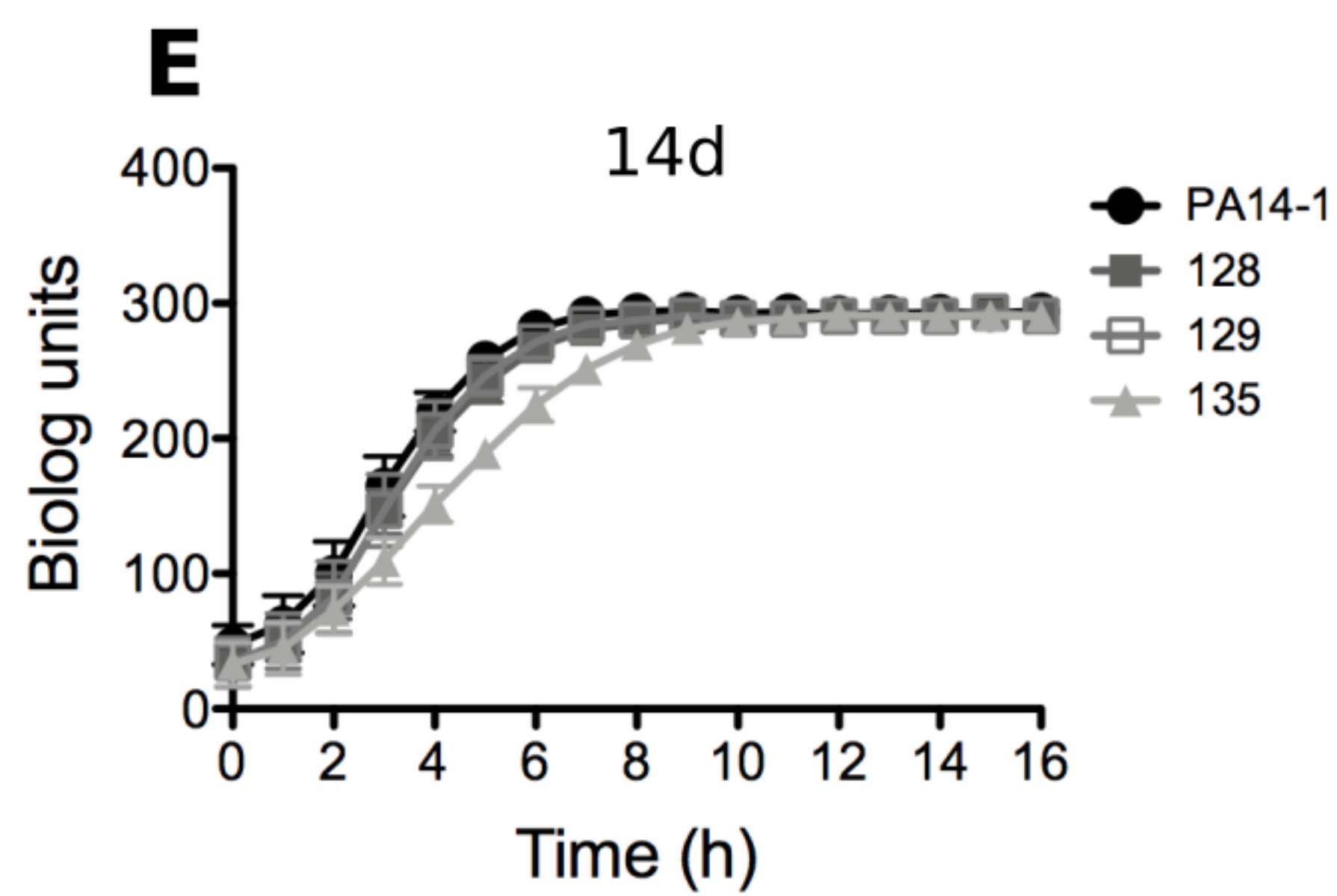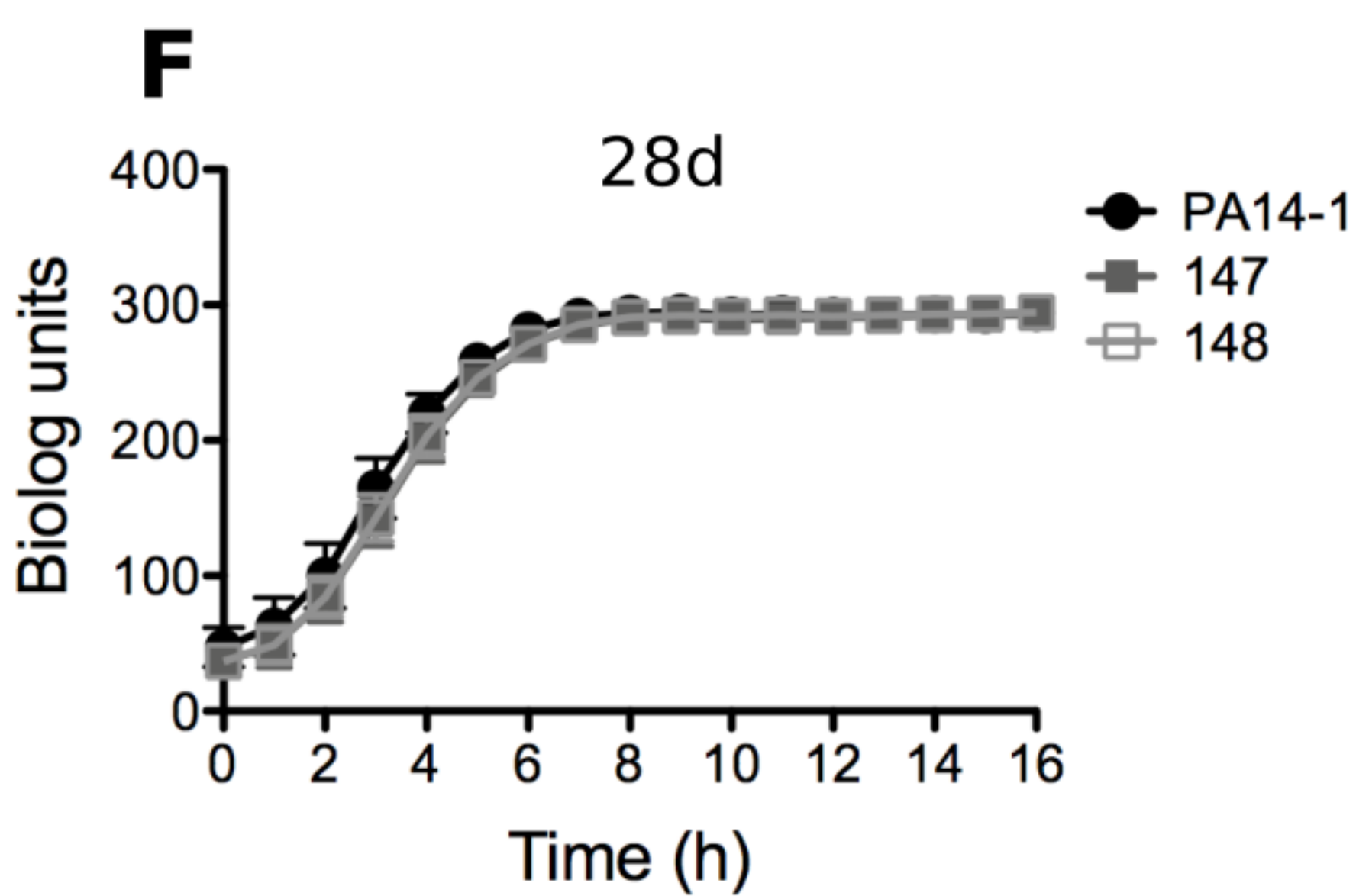

Supplement: FIG S2 [file mBio.01698-19-sf002.pdf]

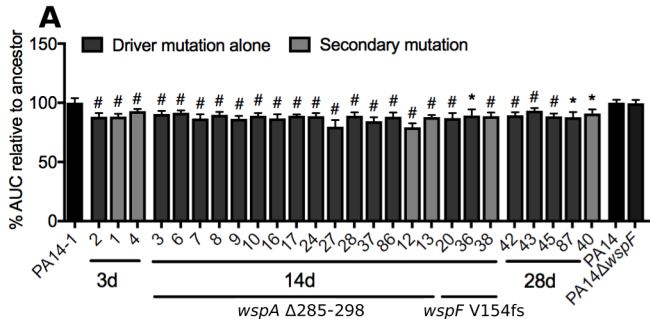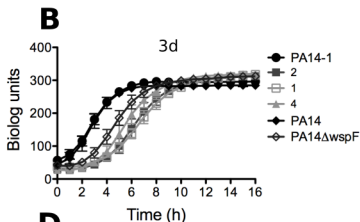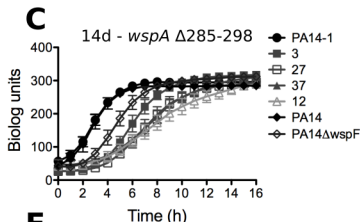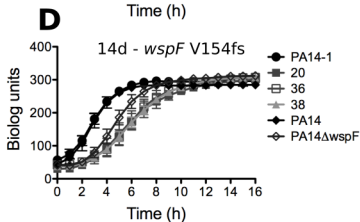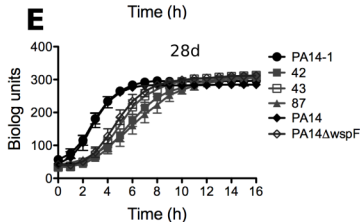

Supplement: FIG S3 [file mBio.01698-19-sf003.pdf]
