# Supplementary material for: Pseudomonas aeruginosa Interstrain Dynamics and Selection of Hyperbiofilm Mutants during a Chronic Infection
Source: mBio. 2019 Aug 13;10(4):e01698-19. doi: 10.1128/mBio.01698-19 (PMC6692513; doi:10.1128/mBio.01698-19)
Supplement: TEXT S1 [file mBio.01698-19-s0001.pdf]

Pseudomonas aeruginosa inter-strain dynamics and selection of hyper-biofilm mutants during a chronic infection

Supplemental Information

Erin S. Gloag, Christopher W. Marshall, Daniel Snyder, Gina R. Lewin, Jacob S. Harris, Alfonso Santos-Lopez, Sarah B. Chaney, Marvin Whiteley, Vaughn S. Cooper, Daniel J. Wozniak

Supplementary Materials and Methods

Bacterial strains and media

P. aeruginosa strains were maintained on LANS (Luria agar no salt; 10g/L tryptone, 5g/L yeast extract solidified with 1.5% agar) unless otherwise specified. RSCV colony morphology was observed on adjusted Vogel-Bonner minimal media (0.2g/L $MgSO_4 \cdot 7H_2O$, 3.5g/L $NaNH_4HPO_4 \cdot 4H_2O$, 10g/L K_2HPO_4, 0.1g/L $CaCl_2$, 2g/L citric acid, 1g/L casamino acid, 40µg/mL Congo red, 15µg/mL brilliant blue, solidified with 1% agar; VBMM).

For *E. coli* strains, 10µg/mL gentamicin, 100µg/mL ampicillin or 15µg/mL tetracycline was used for selection where appropriate. For *P. aeruginosa* strains, 100µg/mL gentamicin, 300µg/mL carbenicillin or 100µg/mL tetracycline was used for selection where appropriate.

Colony morphology

1µL of overnight culture was spotted on VBMM plates and incubated at 37°C for 24h. Colonies were imaged on a Stereo Microscope (AmScope) fitted with a Microscope Digital Color CMOS camera (AmScope). Images were processed in FIJI (1).

Introducing the barcode that the *Tn7* site

Prior to infection, each strain had been tagged with a unique barcode at the Tn7 site on the genome using a genetic barcoding strategy. First, a synthetic insert with the sequence 5’-GTTCAGAGTTCTACAGTCCGACGATCANNNNNNNNNNTGCGCCGTAGTCCCAATGAAAAACCTATGGACTTTGTTTTGGGTAGCATCAGGAATCTGAACCCTGTGAATGTGGGGGTCGCGCGCATAGACCTTTATCTCCGGTTCAAGTTAGGCATGAGGCTGCATGCTACGTTGTCACACCTACACTGCTCGAAGTAAATATGGGAAGCGCGCGGCCTGGCCCGAGGCGTTCCGCGCCGCCACGTGTTCGTTAACTGTTGATTGGTGGCACATAAGCAATACCGTAGTCGGCTAGGTCAAATAGAGTGCTTTGATATCAGCATGTCTAGCTTAGATCGGAAGAGCAC -3’ was constructed using polymerase cycling assembly (PCA) (2). This amplicon consists of the Illumina Read 1 Primer site (underlined), ten random nucleotides (N) which form the barcode, 295 bp of arbitrary sequence, and the Illumina Read 2 Primer site (underlined). To construct this fragment, the oligonucleotides listed in Table S3 were added to a 20 µL reaction containing 0.2 µL Phusion Hot Start II DNA Polymerase (NEB), 4 µL 5x Phusion HF buffer, and 0.4 µL dNTP mix (10 mM each). The reaction was incubated according to the following protocol: 98 °C, 30”; 22 cycles of (98 °C, 10”; 58 °C, 30”; 72 °C, 15”); 72 °C, 5’. The 347 bp band gel purified to create the insert library.

To prepare the plasmid vector, pUC18-mini-Tn7T-Gm (3) was linearized by a *Bam*HI digestion. Gel-purified product was used as a template in a 20 µL PCR reaction containing 0.2 µL Phusion Hot Start II DNA Polymerase (NEB), 4 µL 5x Phusion HF buffer, 0.4 µL dNTP mix (10 mM), and 1 µL of primers KCGbTn7TTSv3F2 and KCGbTn7TTSv3R1 (Table S3) at 10 µM. This PCR was incubated according to the following protocol: 98 °C,

30 sec; 30 cycles of (98 °C, 10 sec; 58 °C, 30 sec; 72 °C, 1 min 30 sec); 72 °C, 5 min. The 4.3 kb product was gel purified to create the PCR-amplified vector.

The insert library and the vector were combined by Gibson assembly (4). The reaction was electroporated into *E. coli* strain DH5α and grown overnight on LA (Luria agar; 10g/L tryptone, 5g/L yeast extract solidified with 1.5% agar) supplemented with 10 ug/mL of gentamicin to yield ~80,000 colonies. A > 200 µg of a high-complexity plasmid library was prepared from the combined colonies. To tag the *P. aeruginosa* strains, 1 ug each of the plasmid library and the transposase-containing plasmid pTNS2 was electroporated into the *P. aeruginosa* strain of interest (3). Electroporants were selected for by growth on LA supplemented with 100 ug/mL of gentamicin.

PCR for identifying the strain frequencies from the wound.

To identify the strain frequencies in the porcine wounds, total DNA was extracted from the tissue as follows. Tissue was added to 2mL Goodman's buffer A (100 mM NaCl, 100 mM Tris-HCl pH 8, 10 mM EDTA pH 8, 3.33% SDS, 0.1% sodium deoxycholate) in a bead beater tube with 0.1mm glass beads and 2-3mm zirconia beads. The tissue was lysed by placing tubes in a TissueLyser II (QIAGEN) for 30s at 50Hz. This was repeated 4 times, placing tubes on ice for 30s in between each lysis round to prevent tubes from overheating. Lysed tissue was incubated with 50µL proteinase K (20mg/mL) for 3h at 55°C. 2mL of phenol, chloroform, isomyl alcohol solution (25:24:1, pH 8) was added and samples were centrifuged for 5min at room temperature. The aqueous phase was removed and mixed with 1.5mL isopropanol. Sample was incubated for 30min at -20°C before being centrifuged for 30min at 4°C. The pellet was washed in 1mL 75% EtOH and again

centrifuged. EtOH was removed and the DNA pellet air-dried and resuspended in 500µL sterile water.

The barcode region at the *Tn7* site was amplified from the extracted DNA by the following PCR using Expand Long-Template Polymerase (Roche). One 50 µL reaction consisted of 0.75 µL polymerase, 5 µl “Buffer 2”, 1 µl 10 mM dNTP mix, 0.5 µL of primers Tn7_F and Tn7_R (Table S3) at 30 µM, 10 µL DNA (20-250 ng), and 31.5 µL water. Cycling conditions were 95 °C, 2’; 30 cycles of (95 °C, 45”; 54 °C, 45”; 68 °C, 1’); 68 °C, 5’. PCR products were confirmed by running 2 µL on a 1% agarose gel with the Gene Ruler 1 kb plus ruler (Thermo Fisher) and quantified with ImageJ (5). This quantification was used to construct pools of PCR products with approximately equal amounts of each PCR product. As our sequencing target size was 425 bp, pools were size selected using a BluePippin with a window of 375-475 bp.

Biolog assay

A Biolog assay was used as a surrogate indicator for metabolic activity. Strains were set up in a 96 well plate and incubated statically overnight at 37°C. Cultures were diluted 1:10 in 100µL LBNS with 1.2% Biolog Dye A in a Biolog 96 well plate. Plates were incubated in an OmniLog incubator (OmniLog) at 37°C for 24h and readings were taken every 15min. Data were processed using the Biolog kinetic software (OL_FM_12), and AUC calculated using the Biolog parametric software (OL_PR_12). Triplicate measurements were performed for each isolate, each in duplicate.

Genome assembly and annotation

In order to conduct homology searches for the CRISPR mutations, a *de novo* assembly of *P. aeruginosa* strain B23-2 was generated. SPAdes version 3.10.1 (6) was used to

assemble the isolate using the command $spades.py –careful -sc --pe1-1 read1_paired.fq.gz --pe1-2 read2_paired.fq.gz --pe1-s read1_unpaired.fq.gz -t 8 -k 21,33,55,77 -o b23_spades_out. The assembly was annotated using Prokka v1.12 (7) with the command $prokka --centre X --force --locustag b23_91 --outdir b23_spades_out --prefix b23_91 --gffver 3 --cpus 8 contigs.fasta.

Homology modeling

The PA14 WspA sequence was obtained from the Pseudomonas genome database (8). The sequence was submitted to SWISS-MODEL using a template search (9). Quality of the returned homology models was assessed on the Global Model Quality Estimation (GMQE) score (numbers closer to 1 indicate more accurate model) and the QMEAN score (numbers closer to 0 indicate that the model is comparable to experimental structures). The homology model of PA14 WspA against the MCP of *Thermotoga maritima* (PDB 3JA6; (10)) was determined to be the most accurate. The homology model had a GMQE score of 0.37 and QMEAN score of -1.68.

Supplementary Results

Non-RSCV PA14-1 populations in the porcine wounds acquire mutations

We were also interested in determining if the non-RSCV PA14-1 population in the porcine wounds acquired adaptive mutations. One biopsy from each wound was spread plated on VBMM supplemented with gent. Five non-RSCV colonies, i.e. those displaying a wildtype colony phenotype, were randomly selected. Therefore, for each timepoint, 20 non-RSCV isolates were randomly selected. The barcode colony PCR was used to identify the ancestor strain of each isolate. Similar to the RSCV sub-population, only PA14-1 and PAO1-B11 isolates were identified (Fig S2A). The two strains showed opposite frequency trends. PA14-1 was dominant on 3-d and then began to decline. In contrast, PAO1-B11

levels on 3-d were low and increased over time to become dominant on 28d (Fig S2A). These observations are in keeping with the strain frequency previously identified (Fig 1B).

Whole genome sequencing was performed to identify if these isolates had non-RSCV adaptive mutations (Table S1). Surprisingly, very few had acquired chromosomal mutations. Two isolates, 108 and 112 had acquired a SNP in the intergenic region between *fabI* and *ppiD*. These are two enzymes involved in fatty acid synthesis. The observation that the SNP occurs in the intergenic region suggests that these two enzymes may have altered expression levels in these isolates (Table S1). Furthermore RSCV-13 and RSCV-4 had the same secondary mutation (Table 3). Finally, isolate 129 had a SNP in PA14_29390 which is annotated as a type VI secretion system protein belonging to the VgrG superfamily. However, this SNP was synonymous and unlikely to affect the protein function (Table S1).

To assess the biofilm formation of these isolates, 24h biofilms were grown in 96 well plates and biomass levels were quantitated by staining with crystal violet. As expected, the PA14-1 non-RSCV isolates had biofilm levels equivalent to the ancestor PA14-1 (Fig S2B). When assessing the metabolic activity of the isolates compared to the ancestor strain, there initially appeared to be a defect based on the area under the curve (AUC) measurements (Fig S2C). However, the kinetic curves of representative isolates were similar to PA14-1, with the exception of isolate 135, which had a reduced exponential phase (Fig S2D-F). It is currently unclear why the growth of this isolate is affected.

Analysis of the CRISPR insertion in PA14-1 RSCV-12 and RSCV-38 isolates

The RSCV-12 CRISPR spacer was identical to the protospacer (corresponding sequence in the infective mobile genetic element (11)) identified in contig-107 from the B23-2

assembly (Fig S4A). RSCV-38 CRISPR spacer had one mismatch between it and the protospacer in contig-95 (Fig S4B). Following both protospacers were GG dinucleotides (Fig S4; underlined text), which is the protospacer adjacent motif (PAM) for type I-F CRISPR-Cas systems that PA14 possesses (12, 13). PAMs are the site on the infective genetic element targeted by the endonuclease of the CRISPR-Cas system (11). A BLAST search of the contig sequences suggests that they align to two different prophages in B23-2. Contig-107 aligns 99% to JBD24 phage, with 1 mismatch, while contig-95 aligns 99% to LPB1 phage, with 3 mismatches. The BLAST search also identified two additional protospacers in contig-107 that aligned to CRISPR spacers in *P. aeruginosa* strains. However, only one possessed the conserved GG PAM (Table S2, Fig S4A; red and blue text). This suggests that the phage that infected RSCV-12 is a prevalent *P. aeruginosa* phage, capable of infecting a wide range of *P. aeruginosa* host strains.

WspA Δ285-298 mutation leads to auto-induction of the Wsp pathway

As the *wspA* Δ285-298 was the most common driver mutation, we investigated how it may lead to elevated c-di-GMP production. We observed that flanking the junctions of this deletion was a direct repeat at 837-854bp and 921-938bp (Fig S5A; bold text (14)).

The WspA Δ285-298 mutation occurs between the predicted HAMP domain (histidine kinases, adenylate cyclases, methyl accepting proteins, and phosphatases) and the signaling domain (SD; Fig S6A). MCP cytoplasmic domains are comprised of consecutive 7aa heptads (15). MCPs are defined into classes based on the number of heptads in the cytoplasmic domain (15), with *P. aeruginosa* WspA belonging to the 40H (40 heptads) MCP class (16). The 285-298aa (14aa) deletion results in complete deletion of heptads N19 and N16 (Fig S6B).

To observe the localization of the deletion in the protein structure and gain insight into how the Δ285-298 mutation may alter the function and signaling of WspA, we generated a homology model of PA14 WspA against the *Thermotoga maritima* MCP (PDB 3JA6; (10)) (Fig S6C). Based on the model, the region of the deletion is predicted to occur opposite the methylation site (Fig S6C). We predict that the deletion could de-stabilize or alter the methylation site resulting in auto-induction of WspA and sustained WspR activation.

References

1. **Schindelin J, Arganda-Carreras I, Frise E, Kaynig V, Longair M, Pietzsch T, Preibisch S, Rueden C, Saalfeld S and Schmid B.** 2012. Fiji: an open-source platform for biological-image analysis. Nature methods **9**:676.

2. **Smith HO, Hutchison CA, Pfannkoch C and Venter JC.** 2003. Generating a synthetic genome by whole genome assembly: ϕX174 bacteriophage from synthetic oligonucleotides. Proceedings of the National Academy of Sciences **100**:15440-15445.

3. **Choi K-H, Gaynor JB, White KG, Lopez C, Bosio CM, Karkhoff-Schweizer RR and Schweizer HP.** 2005. A Tn7-based broad-range bacterial cloning and expression system. Nature methods **2**:443.

4. **Gibson DG, Young L, Chuang R-Y, Venter JC, Hutchison CA and Smith HO.** 2009. Enzymatic assembly of DNA molecules up to several hundred kilobases. Nature methods **6**:343-345.

5. **Schneider CA, Rasband WS and Eliceiri KW.** 2012. NIH Image to ImageJ: 25 years of image analysis. Nat Methods **9**:671-675.

6. **Nurk S, Bankevich A and Antipov D.** 2013. Assembling genomes and mini-metagenomes from highly chimeric reads, 158–170. Research in Computational Molecular Biology, Lecture Notes in Computer Science **7821**.

7. **Seemann T.** 2014. Prokka: rapid prokaryotic genome annotation. Bioinformatics **30**:2068-2069.

8. **Winsor GL, Griffiths EJ, Lo R, Dhillon BK, Shay JA and Brinkman FS.** 2015. Enhanced annotations and features for comparing thousands of Pseudomonas genomes in the Pseudomonas genome database. Nucleic acids research **44**:D646-D653.

9. **Guex N, Peitsch MC and Schwede T.** 2009. Automated comparative protein structure modeling with SWISS‐MODEL and Swiss‐PdbViewer: A historical perspective. Electrophoresis **30**.

10. **Cassidy CK, Himes BA, Alvarez FJ, Ma J, Zhao G, Perilla JR, Schulten K and Zhang P.** 2015. CryoEM and computer simulations reveal a novel kinase conformational switch in bacterial chemotaxis signaling. Elife **4**:e08419.

11. **Barrangou R and Marraffini LA.** 2014. CRISPR-Cas systems: prokaryotes upgrade to adaptive immunity. Molecular cell **54**:234-244.

12. **Zegans ME, Wagner JC, Cady KC, Murphy DM, Hammond JH and O'Toole GA.** 2009. Interaction between bacteriophage DMS3 and host CRISPR region inhibits group behaviors of Pseudomonas aeruginosa. Journal of bacteriology **191**:210-219.

13. **Cady K, White A, Hammond J, Abendroth M, Karthikeyan R, Lalitha P, Zegans M and O'Toole G.** 2011. Prevalence, conservation and functional analysis of Yersinia and Escherichia CRISPR regions in clinical Pseudomonas aeruginosa isolates. Microbiology **157**:430-437.

14. **Starkey M, Hickman JH, Ma L, Zhang N, De Long S, Hinz A, Palacios S, Manoil C, Kirisits MJ, Starner TD, Wozniak DJ, Harwood CS and Parsek MR.** 2009. Pseudomonas aeruginosa rugose small-colony variants have adaptations that likely promote persistence in the cystic fibrosis lung. J Bacteriol **191**:3492-3503.

15. **Alexander RP and Zhulin IB.** 2007. Evolutionary genomics reveals conserved structural determinants of signaling and adaptation in microbial chemoreceptors. Proceedings of the National Academy of Sciences **104**:2885-2890.

16. **Ortega DR, Fleetwood AD, Krell T, Harwood CS, Jensen GJ and Zhulin IB.** 2017. Assigning chemoreceptors to chemosensory pathways in Pseudomonas aeruginosa. Proceedings of the National Academy of Sciences:201708842.

17. **Kim W, Levy SB and Foster KR.** 2016. Rapid radiation in bacteria leads to a division of labour. Nature communications **7**:10508.

18. **Winsor GL, Khaira B, Van Rossum T, Lo R, Whiteside MD and Brinkman FS.** 2008. The Burkholderia Genome Database: facilitating flexible queries and comparative analyses. Bioinformatics **24**:2803-2804.

19. **Norman A, Ciofu O, Amador CI, Høiby N and Jelsbak L.** 2016. Genome sequence of Pseudomonas aeruginosa strain DK1-NH57388A, a stable mucoid cystic fibrosis isolate. Genome announcements **4**:e00008-00016.

Supplementary Figure Legends

Supplementary Figure 1: Phenotypic profile of *P. aeruginosa* strains used in the infection. (A) Metabolic activity of the 6 *P. aeruginosa* strains assayed for 16h at 37°C using a Biolog system. **(B)** Area under the curve (AUC) of the biolog kinetic plot depicted in **(A)**. ** p-value <0.01. **(C)** Biofilms of the 6 *P. aeruginosa* strains were grown for 24h at 37°C in a 96 well plate. Biomass levels were quantified by crystal violet staining. Data depicted as mean ± SD, n=3. **(D)** Frequency of each strain in the starting inoculum determined by sequencing the strain specific barcodes at the *Tn7* site. The proportion of each barcode was expressed as a percentage of the total sequence counts.

Supplemental Figure 2: The PA14-1 non-RSCV population in the porcine wounds behave similar to the ancestor strain. (A) 5 non-RSCV colonies were randomly selected from each wound. The ancestor of each colony was identified and expressed as a percentage of the total number of colonies isolated from that timepoint. **(B)** 24h biofilms were grown in 96 well plates and biomass levels quantified by crystal violet staining. Biomass levels are expressed as a percentage of PA14-1 which was set to 100%. ns indicates no significant difference. **(C)** AUC of Biolog kinetic curves. AUC are expressed

as a percentage relative to PA14-1 which was set to 100%. Gray scale in **(B)** and **(C)** indicate different wounds. **(D-F)** Kinetic Biolog curves of representative non-RSCV PA14-1 isolates from each timepoint (labeled). Data presented as mean ± SD, n=3. * p-value <0.05, ** p-value <0.01, *** p-value <0.001

Supplementary Figure 3: PA14-1 RSCVs from the porcine wounds have an extended lag phase compared to the ancestor strain. (A) AUC of Biolog metabolic kinetic curves of all sequenced PA14-1 RSCVs, expressed as a percentage relative to PA14-1. PA14Δ*wspF* and its parent PA14 were used for comparison. * p-value <0.01, # p-value <0.001 compared to the ancestor strain. **(B-E)** Kinetic Biolog curves of representative PA14-1 RSCVs (labeled). Data presented as mean ± SD, n=3.

Supplemental Figure 4: Contig sequence from B23-2 with the protospacer sequence. There is currently no annotated sequence for B23. Therefore the CRISPR spacer sequences were aligned against the contig sequences of the ancestor B23-2 which was sequenced along with the representative RSCVs. Protospacer for the CRISPR spacer in **(A)** RSCV-12 (contig-107) and **(B)** RSCV-38 (contig-95) were identified in two separate contigs (bold text). For the protospacer sequence in **(B)** contig-95 there was one base pair mismatch when comparing the CRISPR spacer to the protospacer (base pair that is not bold). After each protospacer there is a conserved GG PAM motif (underlined) which is required for type 1-F CRISPR-Cas families which *P. aeruginosa* PA14 possesses. A BLAST search of the contig sequences identified CRISPR spacers in *P. aeruginosa* strains (Table S2) aligned to other protospacers in **(A)** contig-107, indicated by the red and blue text.

Supplementary Figure 5: WspA deletions. (A) 42bp deletion in *wspA* is located between a direct repeat**.** Gene sequence of PA14 *wspA*. Underlined is the 42bp deletion (853-894bp) in the *wspA* mutants. Either side of this sequence is a direct repeat indicated in bold text. **(B)** Alignment of WspA deletions. Deletions in homologous regions of *wspA* have been observed in *in vitro* evolved RSCVs; a 286-307aa deletion in *P. aeruginosa* PAO1 (MJK8) (14), a 284 – 311aa deletion in *P. fluorescence* Pfl01 (17) and a 307-313aa deletion in *Burkholderia cenocepacia* HI2424. The different domains of WspA were determined from Pfam analysis from the Pseudomonas Genome Database (8) and the Burkholderia Genome Database (18). Domains are colored as follows: purple = ligand binding domain or the four helix bundle domain, green = HAMP or linker domain and blue = MCP signaling domain. The region of the deletion in each protein is indicated in red.

Supplementary Figure 6: 14aa deletion in WspA is predicted to occur opposite the methylation site. (A) Schematic of WspA. The different domains of WspA were determined from the Pseudomonas Genome Database Pfam analysis. LBD = ligand binding domain or the four helix bundle domain (3-182aa). HAMP = linker domain (213-261aa). SD = MCP signaling domain (348-505aa). The region of the 14aa deletion is indicated in red (285-298aa). **(B)** The WspA cytoplasmic domain amino acid sequence. The domains are indicated by the same colors in **(A)**. The signaling domain contains two additional features, the kinase interacting subdomain, or 'tip' domain in dark blue (382-420aa) and the predicated methylation site in navy blue (492-501aa) (16). Both the heptad registers (reg) and the heptad number (h#) are labeled (15, 16), with consecutive hetpads indicated in alternating black and grey text. **(C)** Homology model of PA14 WspA modeled against the *T. maritime* MCP (PDB 3JA6; (10)) generated using SWISS-MODEL. Colors correspond to the domains indicated in **(B)**. Model spans 250-541aa of WspA.
