# Supplementary material for: Pseudomonas aeruginosa Interstrain Dynamics and Selection of Hyperbiofilm Mutants during a Chronic Infection
Source: mBio. 2019 Aug 13;10(4):e01698-19. doi: 10.1128/mBio.01698-19 (PMC6692513; doi:10.1128/mBio.01698-19)
Supplement: TABLE S1 [file mBio.01698-19-st001.pdf]

Table S1: Mutations in non-RSCV PA14-1 population

Sample			Mutations	
Day	#	Wound#	Gene	Mutation
3	97	1	No mutations	
	98	1	No mutations	
	99	1	No mutations	
	100	1	No mutations	
	101	1	No mutations	
	102	2	No mutations	
	103	2	No mutations	
	104	2	No mutations	
	105	2	No mutations	
	107	3	No mutations	
	108	3	*fabI/ppiD*	TTC → TCC
	109	3	No mutations	
	112	4	*fabI/ppiD*	TTC → TCC
	113	4	No mutations	
	114	4	No mutations	
	115	4	No mutations	
	116	4	No mutations	
14	127	3	No mutations	
	128	3	No mutations	
	129	3	type VI secretion protein ImpA	H517H (CAT→CAC)
	133	4	No mutations	
	135	4	No mutations	
28	147	3	No mutations	
	148	3	No mutations	
