# Supplementary material for: Pseudomonas aeruginosa Interstrain Dynamics and Selection of Hyperbiofilm Mutants during a Chronic Infection
Source: mBio. 2019 Aug 13;10(4):e01698-19. doi: 10.1128/mBio.01698-19 (PMC6692513; doi:10.1128/mBio.01698-19)
Supplement: FIG S4 [file mBio.01698-19-sf004.pdf]

A

RSCV-12

>91_NODE_107_length_1124_cov_80.5597

TCAGTACCGGACCACCACGTCCGGCAGACCGTCCCGGCCAGGCCGGGGCGGTTTCGCTCA
GTGGACGGTGCGGTTCTGGGCCTTGGTGTAGTGGATTTCCTGCTGCGGTTCCTCGCCGCG
AGCGCGCCGTGCCAGGCACTCGTCGCAGGCGCAGGAAACGGCGTTACCAACTCCGTTCTG
CTTTGCGAGTTCTCGGCCGAGGGACATGGTCGATTGCATAAGGGCGTAGGCGATCTTCGA
TGCTTCGGAGTCGTGCAGCGGCGGGCCTTGCATGGAGAAATCTATTCCGCCTTCGGTGTC
GCTTAGGGTGATGGTGTATACGGCCATGGCTATGCCTCCTTCTGTGCGGATGCCTGCTTT
TCCAGCTGGCGGATGCGGGTGCGAATTTTCTGCTCGACGATCTTCTGGAGGCCGGTGACC
GCGAGCGCGGCGCGGCATTGCTCCAGGTCGAAGCCGGCAACGGCGCGGAGTCGGCTATCG
ACGTCTACGGCGCCGTAGCTCTGTCCGGGGTGATTGAAGGGGTTGGACTGCTGCATTTCA
CACCCCCAGCGCACGGAAGCCGGGCCGATCCTGCTGGCCCAGGGTCTGGATGTACCGGGC
GATGGAGCCCAGGTTTTCGCGGTCGGTGGCGTCGGGGCTCACACCCGGCACGCGCCGGGC
GACTTTCTTGCCGACCTGGTGCGGCTCAGCGGTTGCAAAGACCGCCGCCCGCACGCTGGC
CAAGTCGCCCACGGCAAGGGCGAAGTAGCCCTCGCCTGGGTGTTCCTCCCGCAGCTCGAT
C**AGGCCGTCGATGCGGCACATGGCGAACGTCTT**GGTCATGATCAGAGCCCCTCCAGCAGC
CGAATGGCATCGTGCGTGACCTGGGCCGGAGACTTCCGGCGAGCGCGTGGTGTAGCTGGG
GCCGGCTCGGCCTGCGGCATGACCAATTGACTGGGGCGTACTATCGTCAACTCGACTTCC
GGCACTTCGCCGACGGTGTACTTGAACCCAGCTCCAGAGGCGTAGTCGGCCTGAACCTCT
GCGGCTCGACTCATGATGTCTATCAGCTTGAGTGCATCAGCCTGGGGTAACAGGAAGCGC
TGATAGTCCAAGGTGACGATGCACAGCTGTTGCGCGCGGTTGCG

B

RSCV-38

>91_NODE_95_length_1389_cov_37.628

CAGCGCTCGCAACGGAAGCACCAAATGCCTGATCCCGCATCCACCTCGGCCGGCAGCGCC
GCGCTGCTGAAAATGTTTGGCATCCATATCAGCGCGGGCGCCCTGGCCGCCGCCCTGGGC
TTCCTGGTCCTGTGGCCCAGGACCATGAAAGAGGGGTTTGCCCGGCTGTTCTGCACCATC
GTCGCGTCCAGCGTCTTCGGCCCGATCCTGGTCGTTTACCTGCACTCCAACCGCCCCGAG
CTGTTCGAGTCCGCCCAGGTGGTGGCCGGGCTCTACCAGTTAGAGCCGGCAGTCGGCCTG
CTGTTCGTTTCCGCTCCGCTCCTGGTGATTGCCGGTCTGCCCGCCTGGTGGCTGATCGGG
GCGGCCCTGCGCCTGTTTGAGCGGGACGGCGATTCATGGCTGGGCGCGTTCGCCCAGTGG
GTAAAACGCAAACTGGAGAACACCTGATGGCCCTTCAACCTCGCGGCATCCGCAACAACA
ACCCCGGC**AACATCGT**C**TGGTCGGCACGCAACAACTGGCA**GGGCCAACTCCCCCACGACC
CGCAGATCGAGCCGCGCTTCTGTCGTTTCGACACGGCGCATAACGGCACCCGCGCATTGG
CCAAGCTGCTGCTGAACTACCGCAAGGTCTATGGCCTGCGCACCGTCGAATCGCTGATCG
CACGCTGGGCGCCGTCGAACGAGAACAACACCCGCGCGTATGCCTCGGCCGTGGCCCGAG
CGATGGGAGTTCCGCCGCAGGCCAGCCTGCACCTGGACCAGGCCACCCTGGTCGCCCTGG
TGACCGCCATCATTCACCAGGAAAACGGGCAGCAGCCCTACAGCGCCGAGCAGATCGCCC
AGGCCGTGCGGGAGGTGCTGTGATGCAACGCCCCAGCGGAATCAGCCTCAGCGATCTGTT
CGCGATCTGCCGTGAAGACCCGGCCAACCGATGGCTCTGGATACGCCTCTATCTCCGCGA
CCTGCTGGCCCGCGTCGTGGTTCTGGTCTTCATGGCAATCGGTGCCGCAGGCCTCGCCTA
TGGCCTGGGAGGGGCGTTCGCCTACGGCTTCATGCAGACCGTTGCGTCCTACCAGGTCCA
GCTCAGCGTCGAGAAATCGCCATGACTTGGCGCGTCGGCCTGATTGTCTTTGTGCTCCTG
GTGATGGTCTGGACGGCCGGCTGGTGGGGCGGTCGCGAGGCTGGTCTGTCCGATGGGCGC
GCCGCCTGCGCCGACGCACAGACCCGAGCCTACCGCGACGTCCTGGAGCAATCGGCGACA
CAACTGAAGGCGGTCCAGGACACCAGCGCGGCTCTTTTCCAGCGCCTGGCCCAGCGGGCC
GACAGCGACCAACAAACTACTCGGGAGCTTCGCCATGCCCTGGCCGAAACCGCTGCTGAT
CGCGCTGCC
