# Supplementary material for: Pseudomonas aeruginosa Interstrain Dynamics and Selection of Hyperbiofilm Mutants during a Chronic Infection
Source: mBio. 2019 Aug 13;10(4):e01698-19. doi: 10.1128/mBio.01698-19 (PMC6692513; doi:10.1128/mBio.01698-19)

A

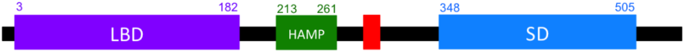

B

```
251 TGFNGMAEELKGLVSQAQRSSVQVTTSVTEIAATSKQQQATATETAATTT 300
reg              abcdefgabcdefgabcdefgabcdefgabcdefgab
h#               N22    N21    N20    N19    N16    N15

301 EIGATSREIAATSRDLVRTMSEVSGAAEQTSTLAGSGQLGLARMEETMHH 350
reg cdefgabcdefgabcdefgabcdefgabcdefgabcdefgabcdefgabc
h#       N14    N13    N12    N11    N10    N09    N08

351 VMGAADLVNAKLAILNEKAGNINQVVTTIVKVADQTNLLSLNAAIEAEKA 400
reg defgabcdefgabcdefgabcdefgabcdefgabcdefgabcdefgabcd
h#      N07    N06    N05    N04    N03    N02    N01

401 GEYGRGFAVVATEVRRLADQTAVATYDIEQMVREIQSAVSAGVMGMDKFS 450
reg efgabcdefgabcdefgabcdefgabcdefgabcdefgabcdefgabcde
h#     C01    C02    C03    C04    C05    C05    C07

451 EEVRRGIAEVGQVGEQLSQIIQQVQALAPRVQMVNEGMQAQATGAEQINQ 500
reg fgabcdefgabcdefgabcdefgabcdefgabcdefgabcdefgabcdef
h#    C08    C09    C10    C11    C12    C13    C14

501 ALVQLGEATGQTVESLRQASFAIDELNLVANGLRNGVSRFKV         542
reg gabcdefgabcdefgabcdefgabcdefgabcdefgabcdef
h#   C15    C16    C19    C20    C21    C22
```

C

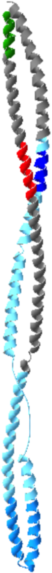

Supplement: FIG S6 [file mBio.01698-19-sf006.pdf]
