# Supplementary material for: Pseudomonas aeruginosa Interstrain Dynamics and Selection of Hyperbiofilm Mutants during a Chronic Infection
Source: mBio. 2019 Aug 13;10(4):e01698-19. doi: 10.1128/mBio.01698-19 (PMC6692513; doi:10.1128/mBio.01698-19)
Supplement: TABLE S2 [file mBio.01698-19-st002.pdf]

Table S2: CRISPR spacers in *P. aeruginosa* strains with a protospacer aligning to contig-107 in B23-2

Strain	CRISPR spacer	PAM[a]	Ref/NCBI #
PA14	AAGGTGACGATGCACAGCTGTTGCGCGCGGTTG	No	CP000438.1 (13)
SMC4515	AAGGTGACGATGCACAGCTGTTGCGCGCGGTTG	No	HQ326226.1 (13)
SMC4500	AAGGTGACGATGCACAGCTGTTGCGCGCGGTTG	No	HQ326214.1 (13)
SMC4499	AAGGTGACGATGCACAGCTGTTGCGCGCGGTTG	No	HQ326213.1 (13)
SMC4497	AAGGTGACGATGCACAGCTGTTGCGCGCGGTTG	No	HQ326212.1 (13)
SMC4493	AAGGTGACGATGCACAGCTGTTGCGCGCGGTTG	No	HQ326209.1 (13)
SMC4486	AAGGTGACGATGCACAGCTGTTGCGCGCGGTTG	No	HQ326202.1 (13)
SMC4485	AAGGTGACGATGCACAGCTGTTGCGCGCGGTTG	No	HQ326201.1 (13)
L10	AAGGTGACGATGCACAGCTGTTGCGCGCGGTTG	No	CP019338.1
M1608	AAGGTGACGATGCACAGCTGTTGCGCGCGGTTG	No	CP008862.2
PA14OR	AAGGTGACGATGCACAGCTGTTGCGCGCGGTTG	No	LT608330.1
Nhmuc	GGCGGTTTCGCTCAGTGGACGGTGCGGTTCT	Yes	CP013479.1
SCVJan	GGCGGTTTCGCTCAGTGGACGGTGCGGTTCT	Yes	CP013478.1
SCVFeb	GGCGGTTTCGCTCAGTGGACGGTGCGGTTCT	Yes	CP013477.1
DK1	GGCGGTTTCGCTCAGTGGACGGTGCGGTTCT	Yes	LN870292.1 (19)

[a] Present/absent after the protospacer in contig-107 (Fig S4A)
