# Supplementary material for: Pseudomonas aeruginosa Interstrain Dynamics and Selection of Hyperbiofilm Mutants during a Chronic Infection
Source: mBio. 2019 Aug 13;10(4):e01698-19. doi: 10.1128/mBio.01698-19 (PMC6692513; doi:10.1128/mBio.01698-19)
Supplement: TABLE S3 [file mBio.01698-19-st003.pdf]

Table S3: Primers used in the study

Primers	Sequence (5' - 3')
Strain identification	
Tn7_F	AATGATACGGCGACCACCGAGATCTACACGTTCAGAGTTCTACAG
Tn7_R[a]	CAAGCAGAAGACGGCATACGAGATxxxxxxGTGACTGGAGTTCAGACGTGTG
KCGbTn7TTSv3F2	AGCTTAGATCGGAAGAGCACACGTCTGAACTCCAGTCACCCCGGGCTGCAGGAATTCCT
KCGbTn7TTSv3R1	TGATCGTCGGACTGTAGAACTCTGAACGTGTAGAACTAGTGAGCTCATGCATGA
Ancestor ID[b]	
PA14-1_F	ATCACAAAAGGACATGC
PAO1-B11_F	ATCAGTGTCGTGGGTGC
B23-2_F	ATCAGCCTATTGTGTGC
CF18-1_F	ATCAGTTACGTCAATGC
MSH10-2_F	ATCATATCAGATTTTGC
S54485-1_F	ATCATTAAACTAGGTGC
AI07-08_R	GCTTATGTGCCACCAATCAACAG
wspA compementation[c]	
wt-wspA_A	taaaacgacggccagtgccaagcttGCGCTGATGGTCGAACTG
wt-wspA_B	gtgacctgcaCCGAAGAACGCTGGGCCT
wt-wspA_C	cgttcttcggTGCAGGTCACCACCTCGG
wt-wspA_D	cagctatgaccatgattacgaattcACTCCGGCGGGAACAGCA
Generation of nucleotide barcodes	
KCGbTn7TagF1	GTTCTACAGTCCGACGATCANNNNNNNNNNTGCGCCGTAGTCCCAATGAAAAACCTATGG
KCGbTn7TagR1	TTCACAGGGTTCAGATTCCTGATGCTACCCAAAACAAAGTCCATAGGTTTTTCATTGGGA
KCGbTn7TagF2	AGGAATCTGAACCCTGTGAATGTGGGGGTCGCGCGCATAGACCTTTATCTCCGGTTCAAG
KCGbTn7TagR2	AGTGTAGGTGTGACAACGTAGCATGCAGCCTCATGCCTAACTTGAACCGGAGATAAAGGT
KCGbTn7TagF3	TACGTTGTCACACCTACACTGCTCGAAGTAAATATGGGAAGCGCGCGGCCTGGCCCGAGG
KCGbTn7TagR3	CACCAATCAACAGTTAACGAACACGTGGCGGCGCGGAACGCCTCGGGCCAGGCCGCGCGC
KCGbTn7TagF4	TCGTTAACTGTTGATTGGTGGCACATAAGCAATACCGTAGTCGGCTAGGTCAAATAGAGT
KCGbTn7TagR4	GTGCTCTTCCGATCTAAGCTAGACATGCTGATATCAAAGCACTCTATTTGACCTAGCCGA

[a] xxxxxx indicates the strain-specific barcode sequence (Table 1).
[b] underlined indicates the strain specific barcode sequence (Table 1).
[c] Lower case is specific to pEX18Ap. Upper case is specific to *wspA*
