# Supplementary material for: Pseudomonas aeruginosa Interstrain Dynamics and Selection of Hyperbiofilm Mutants during a Chronic Infection
Source: mBio. 2019 Aug 13;10(4):e01698-19. doi: 10.1128/mBio.01698-19 (PMC6692513; doi:10.1128/mBio.01698-19)
Supplement: FIG S5 [file mBio.01698-19-sf005.pdf]

A

```
1     GTGAAGAACTGGACTGTTCGCCAGCGAATCCTGGCAAGTTTCGCCGTGATCATCGCGATC 60
61    ATGCTGCTGATGGCCGCTACTGCGTACGTGAAGATGTTGACGGTGGAAAAGGGCGCCTAC 120
121   CGTGTCCAGGACGACGCCATGCCGGGGATGTACTTCATCACCCTGGTACGCAGTTCGTGG 180
181   ACCGACAACTACCTGCAGACCCAGGAACTGTTCGGCGTCACCGACGACCACGAGCTGAGC 240
240   AAGGCCGAGGCTGACAGCATCCTTGCCAGCGAGGAGCGCCTGGACCAGCAGATCGCCTCC 300
301   TACCAGAAGACCATGAACCCGGACGAGGCCCGCGACCACGAACTGCTCGCCGGTTTCCTG 360
361   GGTGTGCGCAAGGCCTACCTGGAGCAGCACGACAAGGTGCTCGAGCTGTATCGCGAGAAA 420
421   CGCTTCGAAGAAGCCGGCAAGCTCGTCGCCGGCCCGTTGACCGAGCACTGGCGCGACGGT 480
481   CGCAAGTACCTCAACGAGCTGATCGAGCTGAACAAGGACATCGCCGATCGCGCCTCGGAG 540
541   AACATCGTCAACGCGGTGGACGACGCCGAACTGAGCATGCTGGTGACCCTGTTGCTGGCG 600
601   GTGGTGGTAGCCGGCATCTGCGGGTTCCTGCTGTTGCGGGCGATCACCCAGCCGATCCAG 660
661   AAGATCGTCCGCAGCCTCGACCTGATGGCCGGCGGCGACCTCACCGCGCGCCTGAACCTT 720
721   GGCCGCCGCGACGAGTTCGGCGCCATCGAGACCGGTTTCAATGGCATGGCCGAGGAACTC 780
781   AAGGGCCTGGTGTCGCAGGCCCAGCGTTCTTCGGTGCAGGTCACCACCTCGGTCACCGAG 840
841   ATCGCCGCGACCTCCAAGCAGCAGCAGGCCACCGCCACCGAAACCGCCGCGACCACCACG 900
901   GAGATCGGCGCCACTTCTCGCGAGATCGCCGCCACCTCGCGCGACCTGGTGCGGACCATG 960
961   AGCGAAGTCTCCGGCGCCGCCGAGCAGACTTCGACCCTGGCCGGTTCCGGCCAGTTGGGC 1020
1021  CTGGCGCGCATGGAGGAAACCATGCACCACGTGATGGGCGCGGCCGACCTGGTCAACGCC 1080
1081  AAGCTGGCGATCCTCAACGAGAAGGCCGGCAACATCAACCAGGTGGTCACCACCATCGTC 1140
1141  AAGGTCGCCGACCAGACCAACCTGCTCTCGCTGAACGCCGCCATCGAGGCGGAGAAGGCC 1200
1201  GGCGAGTACGGCCGCGGGTTCGCCGTGGTGGCCACCGAGGTGCGCCGGCTGGCGGACCAG 1260
1261  ACCGCGGTCGCCACCTACGATATCGAGCAGATGGTGCGCGAGATCCAGTCGGCGGTATCG 1320
1321  GCCGGGGTGATGGGCATGGACAAGTTCTCCGAGGAGGTCCGCCGCGGTATCGCCGAGGTC 1380
1381  GGCCAGGTCGGCGAGCAACTGTCGCAGATCATCCAGCAGGTGCAGGCGCTGGCGCCGCGG 1440
1441  GTGCAGATGGTCAACGAGGGCATGCAGGCCCAGGCGACCGGTGCCGAGCAGATCAACCAG 1500
1501  GCGCTGGTGCAACTGGGCGAGGCCACCGGGCAGACCGTGGAGTCGCTGCGCCAGGCCAGC 1560
1561  TTCGCCATCGATGAGCTGAACCTGGTGGCGAACGGGCTGCGCAACGGCGTATCCCGCTTC 1620
1621  AAAGTCTGA                                                    1629
```

B

```
PA14          MKN------------------WTVRQRILASFAVIIAIMLLMAATAYVKMLTVEKGAYRVQDDAMPGMYFITLVRSSWTDNYLQ 66
PAO1          MKN------------------WTVRQRILASFAVIIAIMLLMAATAYVKMLTVEKGAYRVQDDAMPGMYFITLVRSSWTDNYLQ 66
Pf101         MKN------------------WTLRQRILASFAVIIAIMLLMVVVSYSRLLKIEASENSVRDDAIPGVYYSSMIRGAWVDSYLQ 66
Bcen2424      MATVTPRATNESLHPTIGAARLTLGNRILLSFGVLFVLMLVTAGVSYERLRAINAEAVSIERDSLPGVYLASSLRAAVNETFVM 84
              * .                   *: :*** **.*::.:**: . .:* ::  ::     :. *::**:*  : :*.:  :.::

PA14          TQELFGVTDDHELSKAEADSILASEERLDQQIASYQKTMNPDEARDHELLAGFLGVRKAYLEQHDKVLELYREKRFEEAGKLVA 150
PAO1          TQELFGITDDHELSKAEADSILASEERLDQQIASYQKTMNPDEARDHELLAGFQAVRKDYLEQHDKVLELYREKRFEEAGKLVA 150
Pf101         TQELLGLKEGQGISSEDAADYKAFESRLQEQMANYRGTVVTDEDK--LEFAAFEKYHDAYLKIQDAVLDLHKRNLEADAVKLFH 148
Bcen2424      LQQATFVETDPDAVQRDLAKIADATKQVESLSVDYQASIFRDDDR--ARFATFRGAYDRYLPLLTDAVQRHRGSRDE-AIAAY- 164
               *:   :  .    . :  .     .:::.  ..*: ::  *: :    :* *    . **     .:: :: .    *

PA14          GPLTEHWRDGRKYLNELIELNKDIADRASENIVNAVDDAELSMLVTLLLAVVVAGICGFLLLRAITQPIQKIVRSLDLMAGGDL 234
PAO1          GPLTEHWREGRKYLNEMIELNKDIADRASDNIVNAVDDAELSMLVTLLLAVVVAGICGFLLLRAITQPIQKIVRSLDLMAGGDL 234
Pf101         EQLTPTWYSGRMKLNDIISENKRVADQAMANIDEAVAAAKVSMFVSLVVAVLAAGFCGLLLMRAIMAPMNRIVQILDIMRTGDL 232
Bcen2424      AKVLPGWAEVVQDANIMVQENRKFADQSARVIRESVQDAEVVLVGALTIMLLSALGLGYMLYRSVTVPMARLVEVHDVMRTGNL 248
                :   * .     * ::. *: .**::   * ::*  *:::. :* : :: *   * :* *::  *: ::*.   *:*  *:*

PA14          TARLNLGRRDEFGAIETGFNGMAEELKGLVSQAQRSSVQVTTSVTEIAATSKQQQATATETAATTTEIGATSREIAATSRDLVR 318
PAO1          TARLNLGRRDEFGAIETGFNGMAEELKGLVSQAQRSSVQVTTSVTEIAATSKQQQATATETAATTTEIGATSREIAATSRDLVR 318
Pf101         SSRLNLERKDEFGAVETGFNDMMTELTSLVSQAQRSSVQVTTSVTEIAATSKQQQATATETAATTTEIGATSREIAATSRDLVR 316
Bcen2424      THRLDLRRRDEFGTLETGFNRMADELTELVARAQQSSMQVTTSVAEIAATSREQQATANETAATTTEIGATSREIFATSRDLLR 332
              : **:* *:****::***** *  **. **::**:**:******:******::*****.**************** ******:*

PA14          TMSEVSGAAEQTSTLAGSGQLGLARMEETMHHVMGAADLVNAKLAILNEKAGNINQVVTTIVKVADQTNLLSLNAAIEAEKAGE 402
PAO1          TMSEVSGAAEQTSTLAGSGQLGLARMEETMHHVMGAADLVNAKLAILNEKAGNINQVVTTIVKVADQTNLLSLNAAIEAEKAGE 402
Pf101         TMTEVSTAADQASVLAGSGQQGLARMEDTMHSVMGAADLVNAKLAILNEKAGNINQVVVTIVKVADQTNLLSLNAAIEAEKAGE 400
Bcen2424      TMNEVAGVAEQSATLAGVSQSGLTRMGETMRSVMDAAGSVNAKLAILNEKALNINQVVATITKVADQTNLLSLNAAIEAEKAGE 416
              **.**: .*:*::.*** .* **:** :**: **.**. ************ ******.**.**********************

PA14          YGRGFAVVATEVRRLADQTAVATYDIEQMVREIQSAVSAGVMGMDKFSEEVRRGIAEVGQVGEQLSQIIQQVQALAPRVQMVNE 486
PAO1          YGRGFAVVATEVRRLADQTAVATYDIEQMVREIQSAVSAGVMGMDKFSEEVRRGIAEVGQVGEQLSQIIQQVQALAPRVQMVNE 486
Pf101         YGRGFAVVATEVRRLADQTAVATYDIEQMVREIQSAVSAGVMGMDKFSEEVRRGMAEVQQVGEQLSQIIHQVQALAPRVLMVNE 484
Bcen2424      YGRGFAVVATEIRRLADQTAVATYDIEQTVKEIQSAVSAGVMGMDKFSEEVRRGMLDVQQVGGQLSQIIAEVQTLAPRFQMVNE 500
              ***********:**************** *:************************: :* *** ****** :**:****. ****

PA14          GMQAQATGAEQINQALVQLGEATGQTVESLRQASFAIDELNLVANGLRNGVSRFKV-- 542
PAO1          GMQAQATGAEQINQALVQLGEATGQTVESLRQASFAIDELNLVANGLRNGVSRFKV-- 542
Pf101         GMQAQATGAEQINHALVQLGDASSQTVESLRQASFAIDELSQVAVGLRSGVSRFKV-- 540
Bcen2424      GMQTQANGAEQITQALSQLSEAAQQTAESLRQSSQAIDDLTLVANQLRTSVSRFKVDA 558
              ***:**.*****.:** **.:*: **.*****:* ***:*. **  **..******
```
